# Boron Neutron Capture Therapy Enhanced by Boronate Ester Polymer Micelles: Synthesis, Stability, and Tumor Inhibition Studies

**DOI:** 10.1021/acs.biomac.4c00298

**Published:** 2024-06-07

**Authors:** Wan Yun Fu, Yi-Lin Chiu, Shi-Chih Huang, Wei-Yuan Huang, Fang-Tzu Hsu, Han Yu Lee, Tzu-Wei Wang, Pei Yuin Keng

**Affiliations:** Department of Material Science and Engineering, National Tsing Hua University, Hsinchu City 300, Taiwan

## Abstract

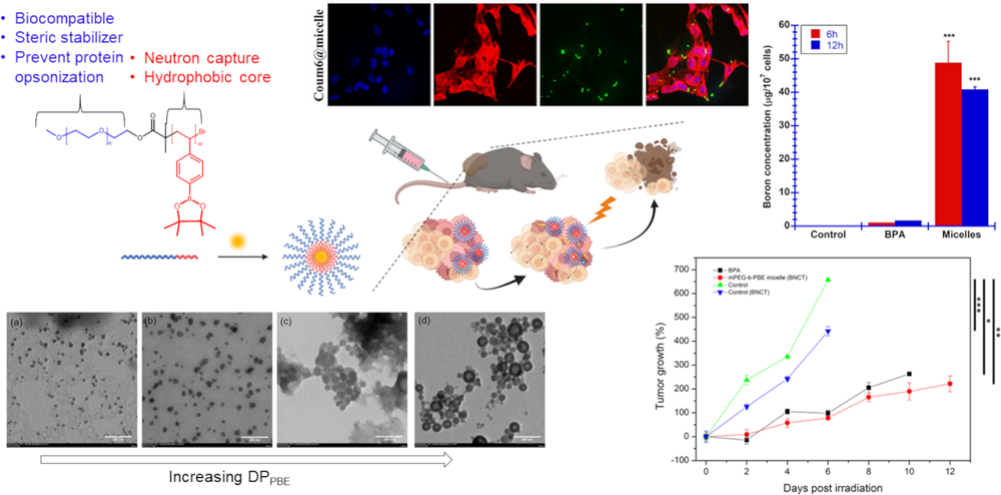

Boron neutron capture therapy (BNCT) targets invasive, radioresistant cancers but requires a selective and high B-10 loading boron drug. This manuscript investigates boron-rich poly(ethylene glycol)-*block*-(poly(4-vinylphenyl boronate ester)) polymer micelles synthesized via atom transfer radical polymerization for their potential application in BNCT. Transmission electron microscopy (TEM) revealed spherical micelles with a uniform size of 43 ± 10 nm, ideal for drug delivery. Additionally, probe sonication proved effective in maintaining the micelles’ size and morphology postlyophilization and reconstitution. In vitro studies with B16–F10 melanoma cells demonstrated a 38-fold increase in boron accumulation compared to the borophenylalanine drug for BNCT. In vivo studies in a B16–F10 tumor-bearing mouse model confirmed enhanced tumor selectivity and accumulation, with a tumor-to-blood (T/B) ratio of 2.5, surpassing BPA’s T/B ratio of 1.8. As a result, mice treated with these micelles experienced a significant delay in tumor growth, highlighting their potential for BNCT and warranting further research.

## Introduction

Boron neutron capture therapy (BNCT) is a promising cancer treatment modality, particularly effective against radioresistant and highly invasive tumors.^1^ This method exploits the unique capability of nonradioactive boron-10 (^10^B) to capture thermal neutrons, leading to a nuclear fission reaction. This reaction generates two high-energy particles: an α particle with an energy of approximately 150 keV μm^–1^ and ^7^Li nuclei with an energy of around 175 keV μm^–1^.^2^ These particles are characterized by high linear energy transfer (LET) and limited path lengths between 4 to 9 μm, effectively confining their ionization energy within the diameter of a single cell.^3^ As a result, BNCT facilitates the targeted destruction of cancer cells enriched with ^10^B, while sparing the surrounding healthy tissues. The success of BNCT critically depends on the tumor-specific uptake of boron-10 agents. Therefore, the development of selective boron delivery agents is of the utmost importance. According to the established criteria for an ideal BNCT agent,^4^ the optimal boron concentration in tumor tissues should range between 20 and 35 μg of ^10^B per gram of tumor tissue, which equates to approximately 10^9^ boron-10 atoms per cell. Additionally, an essential criterion for these agents is achieving a tumor-to-normal tissue ratio exceeding 3:1. This ratio is crucial to ensure targeted destruction of tumor cells, while minimizing damage to adjacent healthy cells. In light of these requirements, contemporary research efforts have been intensively focused on developing boron-10 agents with enhanced tumor-specific accumulation capabilities.

To date, the U.S. Food and Drug Administration has approved only two boron compounds for BNCT clinical trials: sodium borocaptate (BSH) and L-4-dihydroxyboronylphenylalanine (BPA) that are approved by the U.S. Food and Drug Administration for clinical trials.^5,6^ BPA is a derivative of the amino acid phenylalanine, exhibiting selective tumor cell uptake due to the overexpression of the l-amino acid transporters (LAT1) across various cancer types.^7^ This feature endows BPA with a higher selectivity for uptake by tumor cells. Conversely, BSH, a member of the polyhedral boranes, boasts a significantly higher boron content compared to BPA, with approximately 12 times more boron atoms per molecule. BSH enters the tumor cells through passive diffusion across the cell membrane.^7^ However, both BSH and BPA have low molecular weights, which contribute to their short blood circulation half lives and lead to rapid elimination from the systemic circulation.^8,9^ This pharmacokinetic profile limits their effectiveness as boron delivery agents for BNCT. Consequently, the limitations of BSH and BPA have spurred the development of third-generation boron agents. Recent research has shifted toward creating boron-containing compounds that demonstrate high selectivity and intracellular accumulation in tumor cells.^6,10−14^ These efforts aim to meet the stringent criteria for ideal boron delivery agents in BNCT, thereby potentially improving therapeutic outcomes.

Among the third-generation boron drug for BNCT, polymer micelles with a well-defined core–shell structure have entered different phases of clinical trials owing to their high biocompatibility, biodegradability, and their structural resemblance to natural polymer carrier systems such as viruses and lipoprotein.^12,15,16^ Additionally, these micelles offer protective shielding for hydrophobic payloads during in vivo blood circulation, providing favorable pharmacokinetics and in vivo distribution of the drugs at the disease site.^17^ The hydrophilic shell of polymer micelles, being electrically neutral and highly soluble, creates steric repulsion, effectively masking the encapsulated drug and evading the mononuclear phagocytic system (MPS), thus reducing rapid clearance.^18^ Furthermore, the size of polymer micelles, typically within the 10–200 nm range,^19^ exceeds the renal filtration threshold, thereby extending their systemic circulation compared to a small molecular drug that is prone to urinary excretion.^20^ Leveraging the properties of polymeric micelles for delivering a hydrophobic drug across tumor tissues, researchers have explored loading carborane^21,22^ and BSH,^23^ each carrying 12 boron atom per cluster, into these micelles. To overcome the leakage of the hydrophobic boron cluster, researchers have also developed covalent conjugation of boron clusters directly onto polymer micelles. A notable example is Gao and his group’s work, where BSH was attached onto the side chain of poly(chloromethylstyrene) segments, followed by electrostatic self-assembly with polycation containing a radical scavenger, representing a novel approach in BNCT in mitigating potential inflammation upon neutron irradiation.^23^ Other strategies involves synthesizing polymerizable carborane^21,24,24−26^ or conjugating carborane,^24,27,28^ or BSH^23^ onto polymer side chains. These methods result in carborane- or BSH-functionalized polymeric micelles with minimal boron leakage during in vivo blood circulation.

In addition to boron-loaded and conjugated polyethylene glycol-*block*-polylactide block copolymer micelles, the boronic acid containing polymeric micelles have also garnered considerable attention due to their ability of targeting sialic acid receptors expressed on tumor cell membrane^29^ and their stimuli-responsiveness to the changes in pH and glucose concentrations.^30,31^ These polymers can be synthesized through free radical polymerization,^32^ reversible addition–fragmentation transfer (RAFT),^33,34^ atom-transfer radical polymerization (ATRP),^35^ and nitroxide mediated polymerization (NMP).^36^ Common monomers used in these syntheses include 2-acrylamidophenylboronic acid and 4-vinylphenylboronic acid, along with their corresponding boronate ester monomers. Extensive research has explored the use of these boronic acid-containing polymers in a range of applications, including stimuli–responsive drug carriers,^31,34,37^ thermoresponsive hydrogels,^38,39^ HIV-barrier gels,^40^ molecular sensors,^41,42^ cell capture and release,^43,44^ and enzymatic inhibition.^45,46^ Despite these advances, the practical in vivo application of polyboronate ester micelles in boron neutron capture therapy (BNCT) remains an area with limited exploration.^25,30,47−51^

**Scheme 1:**
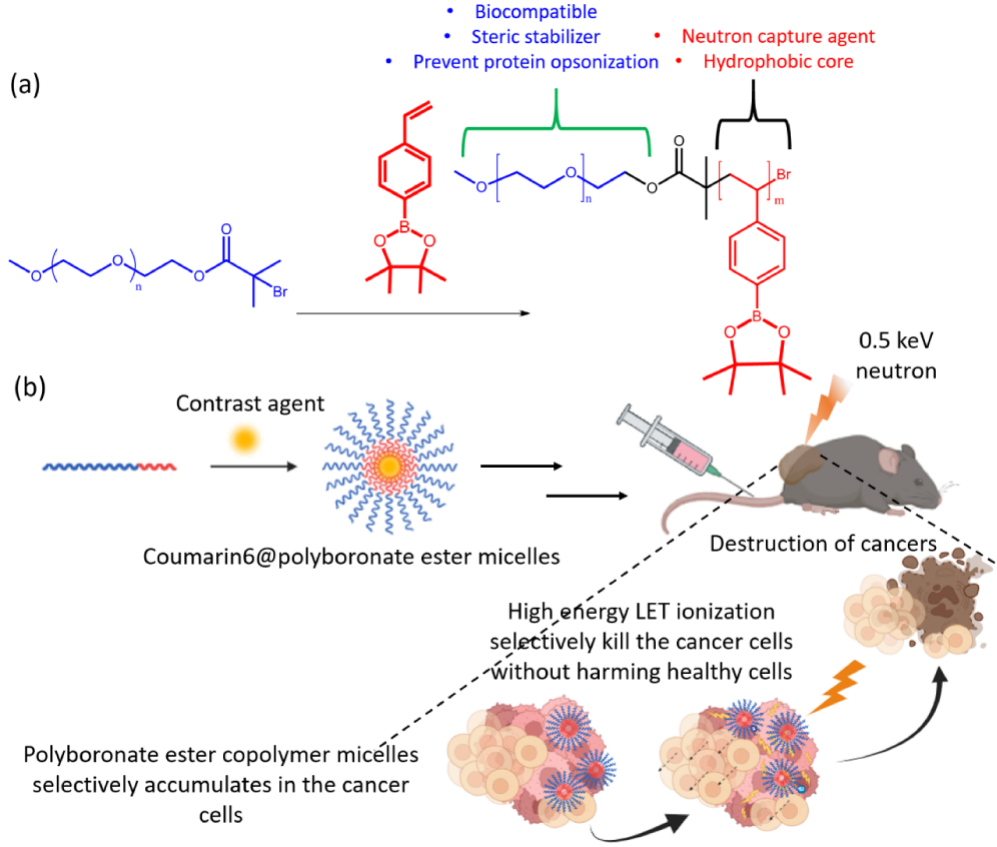
Boron-Rich Polymer Micelles for Boron Neutron Capture Therapy in Cancer Treatment: (a) Synthesis of Poly(ethyleneglycol)-*block*-(poly(4-vinylphenyl boronate ester)) (mPEG-b-PVBE); (b) Co-Assembly of Coumarin-6 Dye within the mPEG-b-PBE_36_ Amphiphilic Block Copolymer for Theranostic Application; the Polyboronate Ester Micelles Demonstrate Selective Accumulation within Melanoma Cells, Facilitating Targeted Ablation of Cancerous Tissue upon Irradiation with Low-Energy Epithermal Neutrons

In response to this research gap, our study focuses on the design and synthesis of a boron-rich amphiphilic block copolymer, poly(ethylene glycol)-*block*-(poly(4-vinylphenyl boronate ester)) (mPEG-*b*-PVBE), using atom transfer radical polymerization (ATRP).^35,52^ This copolymer is developed as a potential neutron capture agent and as a drug nanocarrier for cancer therapy. Our synthesis process involves the preparation of boron-rich polymer micelles through the chain extension of vinylphenylboronate ester (VBE) from a poly(ethylene glycol) methyl ether 2-bromoisobutyrate (mPEG-Br) ATRP macroinitiator, resulting in block copolymers with precisely tunable chain lengths (Scheme 1). The PBE segment of the copolymer plays multiple roles: it acts as a neutron capture agent, serves as the hydrophobic component for encapsulating hydrophobic drugs in combined cancer therapy, and forms the core of the micelles in selective solvents.^53^ Concurrently, the hydrophilic PEG segment provides effective steric stabilization and biocompatibility, aiding in evading opsonization and clearance by the reticuloendothelial system, thus prolonging in vivo circulation time.^54^ This strategic approach exploits the nanoscale properties of polymeric micelles to facilitate high tumor accumulation and uniform distribution within solid tumors while leveraging the high incorporation of B10 into the amphiphilic block copolymers. In this study, we evaluated the efficacy of boron-rich micelles against borophenyl alanine (BPA), the current standard in boron-based drugs, focusing on cellular uptake, tumor accumulation, and antitumor efficacy. The findings reveal that these boron-rich micelles exhibit a 2-fold increase in tumor accumulation and demonstrated a marked prolongation in tumor growth delay compared to the results observed in mice treated with BPA.

The application of polymer micelles as a drug nanocarriers^55−57^ has been extensively explored over recent decades. However, the aspect of their storage stability and formulation for practical application has received comparatively less attention.^55^ A significant challenge in this is the reduced long-term stability of polymer micelles in aqueous media, primarily due to their tendency to aggregate and swell.^58^ Additionally, prolonged storage in aqueous environments poses the risk of microbial and bacterial contamination,^59^ thereby necessitating the implementation of lyophilization or freeze-drying techniques in their development as potential nanodrugs for clinical translation. Freeze-drying emerges as a vital technique in formulation of polymer micelles into a powdered form, increasing their stability during storage and transport for clinical use.^60^ However, the lyophilization process poses its own set of challenges. One critical concern is the maintenance of the structural integrity of polymer micelles, which often suffer from stress-induced damages during lyophilization,^61^ complicating the subsequent reconstitution process. Common strategies to mitigate these issues include the addition of excipient, such as lactose at high concentrations, to aid the dissolution of the powdered polymer micelles in water.^62^ Another approach involves using *tert*-butanol in a one-step freeze-drying process, as demonstrated in the preparation of polyvinylpyrrolidone-*block*-poly(d,l-lactide) (PVP-*b*-PDLLA) block copolymer micelles.^63^ However, this latter strategy is not universally applicable, specifically to PEG copolymers, due to the poor solubility of poly(ethylene glycol) in *tert*-butanol.

Given the limitations of these methods, which require the addition of either excipients or organic solvents, our study systematically investigates a simple and universal reconstitution strategy for lyophilized polymer micelles without the addition of excipients. Addressing these challenges is crucial for the successful translation of polymer micelles into effective nanodrugs for clinical applications. This multifaceted approach, which includes optimizing storage and reconstitution methods and leveraging the unique structural and therapeutic properties of boron-rich polymer micelles, offers a promising pathway in the advancement of targeted cancer therapy.

## Materials and Methods

Poly(ethylene glycol) methyl ether 2-bromoisobutyrate (MW 5000 g/mol) was procured from Sigma-Aldrich and used as received. Dichloromethane (DCM) and anhydrous DCM were sourced from DUKSAN and Sigma-Aldrich, respectively. Triethylamine (TEA) and 2-bromoisobutyryl bromide were acquired from Sigma-Aldrich. 4-Dimethylaminopyridine (DMAP) was purchased from Matrix Scientific. 12 M hydrochloric acid (HCl) was purchased from Honeywell, and ethyl ether was obtained from J.T. Baker. Magnesium sulfate (MgSO_4_) was supplied by Thermo Fisher Scientific. Tetrahydrofuran (THF) used in this study was purchased from Duksan. Acros Organics provided 4-vinylphenylboronic acid and pinacol. Copper(I) bromide (CuBr) and toluene were obtained from Sigma-Aldrich and Alfa Aesar, respectively. PMDETA was supplied by Acros Organics. Aluminum oxide was obtained from Sigma-Aldrich, and *n*-hexane was provided by Aesar. Coumarin 6 was sourced from Sigma-Aldrich. For the biological assay, the MTS reagent (3-(4,5-dimethylthiazol-2-yl)-5-(3-carboxymethoxyphenyl)-2-(4-sulfophenyl)) was acquired from Promega Cooperation. FAST DiI solid and DAPI (4′,6-diamidino-2-phenylindole, dihydrochloride) were supplied by Thermo Fisher Scientific. The HMGB1 ELISA kit was manufactured by Elabscience. TNF-α, IFN-γ, IL-1β, and IL-2 ELISA kits, along with the FITC antimouse CD3 antibody, APC/Cyanine7 antimouse CD8a antibody, and PE antimouse CD4 antibody, were sourced from BioLegend. The calreticulin polyclonal antibody (CRT) was sourced from MyBioSource, and the anti-*g*-H2AX antibody was obtained from BioLegend.

Nuclear magnetic resonance (NMR) spectra were acquired by using a VARIAN VNMRS-700 instrument. Fourier-transform infrared spectroscopy (FTIR) analyses employing the attenuated total reflectance (ATR) technique were conducted using a Bruker Vertex 80v instrument to obtain the infrared spectra. The molecular weight (*M*_w_) and number-average molecular weight (*M*_n_) of the mPEG-*b*-PBE_36_ copolymer were determined using gel permeation chromatography (Waters, USA). The morphology and size of the copolymer micelles were analyzed and measured using a high-contrast transmission electron microscope (Hitachi HT7700 TEM, Japan). The hydrodynamic size of the copolymer micelles was analyzed by using the dynamic light scattering (Malvern Zetasizer Nano ZS90) instrument. After the micelle preparation, the mPEG-*b*-PBE_36_ copolymer micelles were lyophilized using a laboratory freeze dryer (Kingmech, New Taipei City, Taiwan). The lyophilized samples were subsequently reconstituted using a probe sonicator (QSonica Q125, 125 W, 20 kHz, Newtown, CT. USA). Cell viability was assessed by measuring the absorbance using a Molecular Devices SpectraMax 340PC Microplate Reader. To determine the boron content in the samples, they were prepared in a solution of 10 μL hydrofluoric acid (HF) and 1 mL nitric acid (HNO_3_). The analysis was then conducted using inductively coupled plasma-mass spectrometry (ICP-MS, Thermo Fisher Scientific iCAP TQ, Germany). Finally, the cellular uptake of the copolymer micelles was qualitatively examined using a laser scanning confocal microscope (Carl Zeiss LSM780). This analysis provided insights into the distribution of the micelles within cancer cells.

### Synthesis of the ATPR Macroinitiator, Poly(ethylene glycol) Monomethyl Ether 2-Bromoisobutyrate (mPEG-Br)

In a dry 500 mL round-bottom flask equipped with a magnetic stir bar, was charged with monomethyl ether polyethylene glycol (CH_3_(OCH_2_CH_2_)_113_–OH,mPEG, *M*_n_ = 5000 g mol^–1^, 25.0 g, 5.0 mmol) and 400 mL of dichloromethane.^64,65^ The polymer was first dissolved in anhydrous DCM. This was followed by the addition of anhydrous triethylamine (TEA, 2.8 mL, 20 mmol) and 4-dimethylaminopyridine (DMAP, 2.4 g, 20 mmol). The resultant mixture was subsequently cooled in an ice bath, and the temperature was maintained at 0 °C. Meanwhile, 2-bromoisobutyryl bromide (2.5 mL, 20 mmol) was mixed with 50 mL of anhydrous DCM, and this solution was added dropwise to the reaction mixture while stirring continued. After the completion of this addition, the mixture was first treated with diluted hydrochloric acid (HCl), followed by extraction DCM thrice.^66^ The lower layer was collected, and the residual solvent was removed using a rotary evaporator. The concentrated mixture was then precipitated in cold ethyl ether to yield the crude macroinitiator, mPEG-Br, which was dried in an oven thermostated at 40 °C for 48 h. The final product was obtained as a white powder in a yield of 92%. ^1^H NMR (700 MHz, 25 °C, CDCl_3_): δ 3.48–3.78 (br, −OCH_2_CH_2_O−), δ 3.36 (s, 3H, −OCH_3_), 1.92 (s, 6H, −CBr(CH_3_)_2_).

### Synthesis of 4,4,5,5-Tetramethyl-2-(4-vinylphenyl)-1,3,2-dioxaborolane (MBpin)

To prepare an anhydrous solvent, approximately two spatula tips of magnesium sulfate (MgSO_4_) were added to 300 mL of THF and stirred for 10 min.^67^ Following this, MgSO_4_ was filtered out. Subsequently, 4-vinylphenylboronic acid (6.00 g, 40.6 mmol) and pinacol (4.8 g, 40.8 mmol) were dissolved in the anhydrous THF in a 500 mL round-bottom flask. This mixture was stirred at room temperature for 2 h. The resultant solution was then concentrated under reduced pressure using a rotary evaporator. The final product was obtained as a yellow, viscous liquid with a yield of 95%. ^1^H NMR (700 MHz, 25 °C, CDCl_3_): δ 7.67 (d, 2H, *J* = 7.63 Hz, Ar–H), 7.29 (d, 2H, *J* = 7.77 Hz, Ar–H), 6.60 (dd, 1H, *J*_1_ = 10.90 Hz, *J*_2_ = 17.50 Hz, −Ar–CH=CH_2_), 5.69 (d, 1H, *J* = 17.64 Hz, −CH=CH_2_–E), 5.16 (d, 1H, *J* = 10.90 Hz, −CH=CH_2_–Z), 1.22 (s, 12H, Ar–BO_2_(C(CH_3_)_2_)_2_).

### Synthesis of Polyethylene Glycol-*b*-poly(vinylphenyl boronate ester) (mPEG-*b*-PBE_36_) via ATRP

A standard ATRP chain extension procedure of MBpin from the mPEG-Br macroinitiator was employed to synthesize mPEG-*b*-PBE_36_. First, mPEG-Br (1.5 g, 0.3 mmol) was added to a Schlenk flask containing a magnetic stir bar. The flask was sealed with a rubber septum and subjected to degassing for 30 min. Separately, in a 20 mL sample vial also equipped with a magnetic stir bar, CuBr (43.0 mg, 0.3 mmol) was added, sealed with a rubber septum, and underwent vacuum/nitrogen purge cycle three times. MBpin (6.9 g, 30 mmol) was then added into the Schlenk flask using an airtight syringe, followed by purging with nitrogen for 30 min. Subsequently, degassed toluene (8.0 mL) and PMDETA (62.7 μL, 0.3 mmol) were added to the CuBr-containing sample vial using an airtight syringe. This mixture was then transferred to the Schlenk flask. The reaction mixture was incubated under a 100 °C oil bath for 24 h. Upon completion, the reaction was quenched by cooling the mixture to room temperature using cold water. The Schlenk flask was then opened to expose its content to air. The reaction product was dissolved and diluted with tetrahydrofuran, and the catalyst was removed by filtration through an alumina column. The final step involved precipitating the sample with excess cold *n*-hexane and drying it in a 40 °C oven for 48 h. The resultant product appeared as a white powder solid. ^1^H NMR (700 MHz, 25 °C, CDCl_3_): δ 7.20–7.61 (br, Ar–H), 6.02–6.80 (br, Ar–H), 3.40–3.69 (br, −OCH_2_CH_2_O−), 3.29 (s, 3H, −OCH_3_), 0.94–1.46 (br, Ar–BO_2_(C(CH_3_)_2_)_2_).

### Micelle Preparation

#### Method 1

To prepare the micelle solution, 19 mg of amphiphilic mPEG-*b*-PBE_36_ polymer (*M*_n_ = 13 441 g mol^–1^, 1.4 × 10^–3^ mmol) was dissolved in 3 mL of THF under stirring conditions until the solution became transparent. This solution was then added dropwise to 30 mL of deionized (DI) water in a sonication bath and sonicated for 10 min. Subsequently, the mixture was stirred in a fume hood to facilitate the evaporation of THF. After overnight stirring at room temperature, the micelle solution concentration was determined to be 0.6 mg/mL. The solution was gradually frozen to −20 °C and then subjected to lyophilization, yielding a white powder as the final product with a yield of 60%.

#### Method 2

In Method 2, 120 mg of mPEG-*b*-PBE_36_ copolymer (*M*_n_ = 13 441 g/mol) was dissolved in 2 mL of THF. The solution was stirred until clarity was achieved.^31,68^ This solution was then added dropwise to 4 mL of water while undergoing probe sonication. The mixture was transferred to a 20 mL sample vial, and THF was allowed to evaporate overnight in a fume hood. Postevaporation, the sample was vacuumed for 20 min to ensure complete removal of the solvent, resulting in a final copolymer concentration in the aqueous solution of 30 mg/mL. Following the removal of THF, 2 mL aliquots of the copolymer solution (concentration: 30 mg/mL) were diluted with 10 mL of water to obtain a final copolymer concentration of 5 mg/mL. This solution was then slowly frozen to −20 °C and lyophilized, resulting in a white powder with a yield of 70%.

### Encapsulation of Coumarin-6 within the mPEG-*b*-PBE_36_ Micelles (Coum6@micelles)

The encapsulation of Coumarin-6 within the mPEG-*b*-PBE_36_ micelles was carried out using a procedure similar to that previously described, Method 2, allowing for the encapsulation of Coumarin 6 into the polymer micelles during the self-assembly process, thereby resulting in its encapsulation within the micellar structure. Specifically, 120 mg of mPEG-*b*-PBE_36_ block copolymer was dissolved in 2 mL of THF. Concurrently, 20.8 mg of coumarin 6 was dissolved in 0.5 mL of THF. The two solutions were then mixed and stirred thoroughly for 10 min. Subsequently, this mixture was then left in a fume hood overnight to allow for the evaporation of THF. Afterward, the sample was vacuumed for 20 min to ensure complete removal of the solvent. The solution was then diluted to a concentration of 5 mg/mL and centrifuged for 50 min to remove excess dye. Finally, the sample was slowly frozen to −20 °C and subjected to lyophilization, yielding a yellow powder as the final product.

### Reconstitution of Lyophilized mPEG-*b*-PBE_36_ Micelles

#### Reconstituted via Bath Sonication

Deionized (DI) water was added to the lyophilized micelle powder, and the mixture was gently agitated to ensure complete dissolution.^68^ The process yielded a solution with a concentration of 1 mg/mL. The solution was then placed in an ultrasonic bath sonicator (80 W, 40 kHz) and subjected to sonication for 10 min to facilitate reconstitution.

#### Reconstituted by Probe Sonication

DI water was similarly added to the lyophilized micelles, followed by gentle shaking to dissolve the sample, resulting in a 1 mg/mL solution. For reconstitution, a probe sonicator (125 W, 20 kHz) was employed. The sonication process was conducted in intervals of 6 s, accumulating to a total duration of 30 s. Between intervals, the solution was mixed thoroughly with a pipet to ensure uniformity before resuming sonication.

### Stability Test of mPEG-*b*-PBE_36_ Micelles

To evaluate the stability of mPEG-*b*-PBE_36_ micelles, they were incubated in phosphate buffered saline (PBS) and Dulbecco’s modified Eagle medium (DMEM) over a period of 7 days. The experimental protocol was akin to the previously described probe sonicator reconstitution method with the exception that PBS and DMEM, supplemented with 10% fetal bovine serum (FBS), were utilized in place of DI water. Briefly, the lyophilized micelles were dissolved in 1 × PBS or DMEM and reconstituted using a probe sonicator for 30 s. The resultant micelle solution was then left at room temperature for designated time points of 1, 3, and 7 days. Subsequently, the size was measured by using DLS.

### Cytotoxicity Assessment of mPEG-*b*-PBE_36_ Micelles on B16–F10 Melanoma Cells

To evaluate the cytotoxicity of mPEG-*b*-PBE_36_ micelles, we performed an MTS assay on B16–F10 melanoma cells. First, a cell count was performed. The cells, postcentrifugation, were suspended in DMEM. A 20 μL portion of this cell suspension was then mixed with 180 μL of trypan blue, yielding a 10-fold diluted mixture. Subsequently, 20 μL of this diluted mixture was transferred to a cell counting plate. The counting chamber was covered with a cover glass, and the cells were observed and counted under a 100× optical microscope.

In preparation for the assay, 7 × 10^3^ cells/well of B16–F10 melanoma cells were seeded in a 96-well plate and incubated for 24 h to facilitate complete adherence. The micelle solution, initially prepared at a concentration of 1 mg/mL in DMEM, was reconstituted by undergoing 30 s of sonication using a probe sonicator. A series of six centrifuge tubes, each containing 2 mL of DMEM, was prepared. The micelle solution was sequentially diluted to achieve concentrations of 1000, 500, 250, 125, 62.5, 31.25, and 15.625 μg/mL. The existing medium in the 96-well plate was replaced with these varying concentrations of the micelle solutions. The cells were then incubated at 37 °C for 24, 48, and 72 h. At each time point (24, 48, and 72 h) 20 μL of the MTS reagent was added to each well. After a 2 h reaction period, the absorbance at 490 nm was measured to determine cell viability. Cell viability was calculated using eq 1.
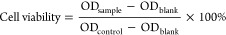
1

### Quantification of Cellular Uptake of Micelles

For the assessment of micelle uptake by cells, 3 × 10^5^ B16–F10 melanoma cells/well were seeded in a 6-well plate. The cells were cultured in 2 mL/well DMEM for 24 h to facilitate cell adhesion. Subsequently, the cells were treated with micelle solutions reconstituted to a concentration of 1000 μg/mL, and for comparative purposes, with 1000 μg/mL BPA. After 6 and 12 h of incubation, the treatment media were removed, and the cells were washed twice with 2 mL of PBS buffer. Next, 1 mL of trypsin was added to each well and allowed to react for 5 min. To neutralize the trypsin and facilitate cell detachment, 1 mL of DMEM was added. The cells were then washed twice with 2 mL of PBS buffer until they were completely rinsed. After washing, the cells were collected and centrifuged at 1600 rpm for 5 min. The supernatant was discarded, leaving behind the cell pellet. This cell pellet was then mixed with 1 mL of concentrated nitric acid and hydrochloric acid in preparation for inductive coupled plasma-mass spectrometry (ICP-MS) for boron quantification.^69^

### Confocal Laser Scanning Microscopy (CLSM) Imaging

To prepare for CLMS imaging, sterilized glass slides were placed within a 24-well plate. A density of 2 × 10^4^ B16–F10 melanoma cells/well was seeded onto these glass slides. The cells were incubated with 1 mL/well DMED for 24 h to facilitate their adhesion to the glass surface. Following this, the cells were treated with 1000 μg/mL reconstituted coum6@micelle solution and incubated at 37 °C for 6 and 12 h. Postincubation, the treatment solution was then removed, and the cells were washed twice with 1 mL of PBS buffer. Subsequently, the cells were fixed using 300 μL/well of formalin for 10 min. To permeabilize the cells, 500 μL/well 0.1 wt % Triton X-100 was added for 5 min. Thereafter, staining was conducted sequentially using FastDil (for cell membrane staining) and DAPI (for nuclear staining), with each reaction allowed to proceed for 20 and 5 min, respectively, under dark conditions.^70^ Upon completion of the staining reactions, the glass slides were removed from the wells. The slides were then sealed with a fluorescent mounting medium to prevent moisture loss in the samples. The prepared samples were stored at 4 °C. Finally, the cellular uptake of the micelles was visualized and analyzed by using confocal microscopy.

### Thermal Neutron Irradiation Experiments

The in vitro BNCT experiments were performed at the Tsing Hua Open-pool Reactor (THOR) following established protocols in the literature.^71−73^ In a typical experiment, 3 × 10^5^ B16–F10 melanoma cells per well were suspended in 2 mL of DMEM and seeded in a 6-well plate. After a 24-h incubation period, the cells were treated with either 1 mg/mL of the reconstructed micelle solution, prepared using DMEM, or a 1 mg/mL BPA solution, which were prepared by diluting a stock solution of 25 mg/mL BPA in DMEM. These incubations were conducted for 6 and 12 h at 37 °C. Postincubation, the medium containing the boron drugs was aspirated and discarded. The cells were washed twice with 2 mL of PBS buffer to remove any free drugs. Subsequently, 1 mL of trypsin was added to detach the cells, followed by a 5 min incubation. The reaction was terminated by adding 1 mL of DMEM, and the cells were washed again. After the final wash with 2 mL of PBS buffer, the cells were centrifuged at 1600 rpm for 5 min. The supernatant was discarded, and the resultant cell pellet was collected. The cells were resuspended in 1 mL of DMEM and transferred into cells cryovials for neutron irradiation. A maximum of four vials were irradiated per session at the THOR. Neutron irradiation was performed in the BNCT clinic at the THOR under the conditions of 1.2 MW, at a neutron flux of 1 × 10^9^ neutrons/cm^2^ s, for a continuous duration of 30 min. Immediately following irradiation, the cells were reseeded back into a 96-well plate at a density of 7 × 10^3^ B16F10 melanoma cells per well and incubated for 24 and 48 h at 37 °C. Subsequently, cell viabilities were assessed using the MTS reagent. For this purpose, 20 μL of the MTS reagent was added to each well, and the absorbance at 490 nm wavelength was measured using a microplate reader.

### In Vivo Tumor Model Establishment

The experimental procedures involving animals were conducted in compliance with the guidelines set by the Institutional Animal Care and Use Committee (IACUC), with approval obtained under reference number 112002. To establish the B16–F10 melanoma mouse model, 4-week-old male C57BL/6JNarl mice were employed. The B16F10 melanoma cells were cultured under a controlled environment of 37 °C and 5% CO_2_ in DMEM supplemented with 10% fetal bovine serum (FBS). Cell harvesting was performed during the exponential growth phase with cell viability and quantification using the trypan blue exclusion assay. For the development of the tumor model, 1 × 10^6^ cells, suspended in 50 μL of PBS were subcutaneously injected into the right hindlimb region utilizing a 27-gauge syringe. The progression and dimensions of the tumors were regularly monitored by using calipers. The tumor volume was calculated using eq 2:
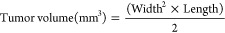
2

In adherence to the euthanasia timing and criteria established by the IACUC, humane euthanasia was performed on animals exhibiting signs of health impairment. This included criteria such as a weight loss exceeding 20–25%, signs of infection, a complete loss of appetite for than 24 h, or when the tumor size exceeding 1000 mm^3^.

### In Vivo Biodistribution of the PEG-*b*-PBE Micelles and BPA

Seventh-day after tumor postinoculation, the tumors reached a measurable size, approximately 100 mm^3^, as determined using calipers. The mice were then randomly divided into two groups, each consisting of five mice (*n* = 5). Both BPA and mPEG-*b*-PBE_36_ micelles were administered intravenous (i.v.) injection at a dosage of 100 mg/kg. The injections were administered every 2 days, for a total of three doses. 24 h following the final dose, the mice were humanely euthanized in accordance with approved ethical guidelines. Subsequent to euthanasia, vital organs including the heart, liver, spleen, lungs, kidneys, and blood, and tumor tissues, were harvested and immediately weighed. All collected samples were treated with a digestion solution composed of 68% nitric acid and hydrofluoric acid. This was followed by an overnight incubation at room temperature to ensure complete digestion of the samples. The boron concentration within these tissue samples was quantitatively analyzed using inductively coupled plasma mass spectrometry (ICP-MS).

### In Vivo Thermal Neutron Irradiation Experiment

In this in vivo thermal neutron experiment, we employed the B16–F10 melanoma tumor model as outlined in the In Vivo Tumor Model Establishment section. Seven days postinoculation, the tumors attained an average volume of 100 mm^3^, as measured with a caliper. Subsequently, the mice were randomly divided into four groups, each comprising five mice (*n* = 5): (1) control, (2) control (BNCT), (3) BPA (BNCT), and (4) mPEG-*b*-PBE_36_ micelles (BNCT). The designation of “BNCT” within parentheses signifies that groups that received neutron irradiation, while its absence indicates groups that did not undergo irradiation.

Each mouse was administered intravenous (i.v.) injections of either BPA or mPEG-*b*-PBE_36_ micelles at a dose of 100 mg/kg every 2 days, resulting in a total of three injections. Following a 24 h interval after the final injection, the mice were immobilized with adhesive tape, securing their body and tail. They were then carefully placed in a custom-designed acrylic holder to ensure that the right hindlimb was centrally located for optimal exposure to neutron radiation. A polyethylene (PE) board was strategically placed atop the holder to achieve the production of epithermal neutron (0.5 eV–10 keV). The tumors were exposed to a neutron flux of 1 × 10^9^ neutrons/cm^2^ and an irradiation power of 1.2 M for 30 min.

### Immunohistochemistry Staining

For immunohistochemistry staining, tumor tissues underwent paraffin-embedding and were sectioned into 5 μm thick. These sections were stained with primary antibodies, specifically targeting caspase-3 (catalog number 19677–1-AP, Proteintech) and p53 (catalog number ab183544, Abcam). The staining process was followed by horseradish peroxidase (HRP)/diaminobenzidine (DAB) detection, employing the Pierce peroxidase immunohistochemistry (IHC) detection kit (catalog number 36000, Thermo Fisher), in accordance with the manufacturer’s guidelines.

### Statistical Analysis

The evaluation of significant differences between groups was conducted using the unpaired Student’s *t* test for comparison of two groups within the context of individual experiments. The levels of statistical significance were set as follows: *p < 0.5 for indicative significance, **p < 0.05 for moderate significance, and ***p < 0.005 for high significance.

## Results and Discussion

### Synthesis and Characterization of mPEG-*b*-PBE_36_

**Figure 1:**
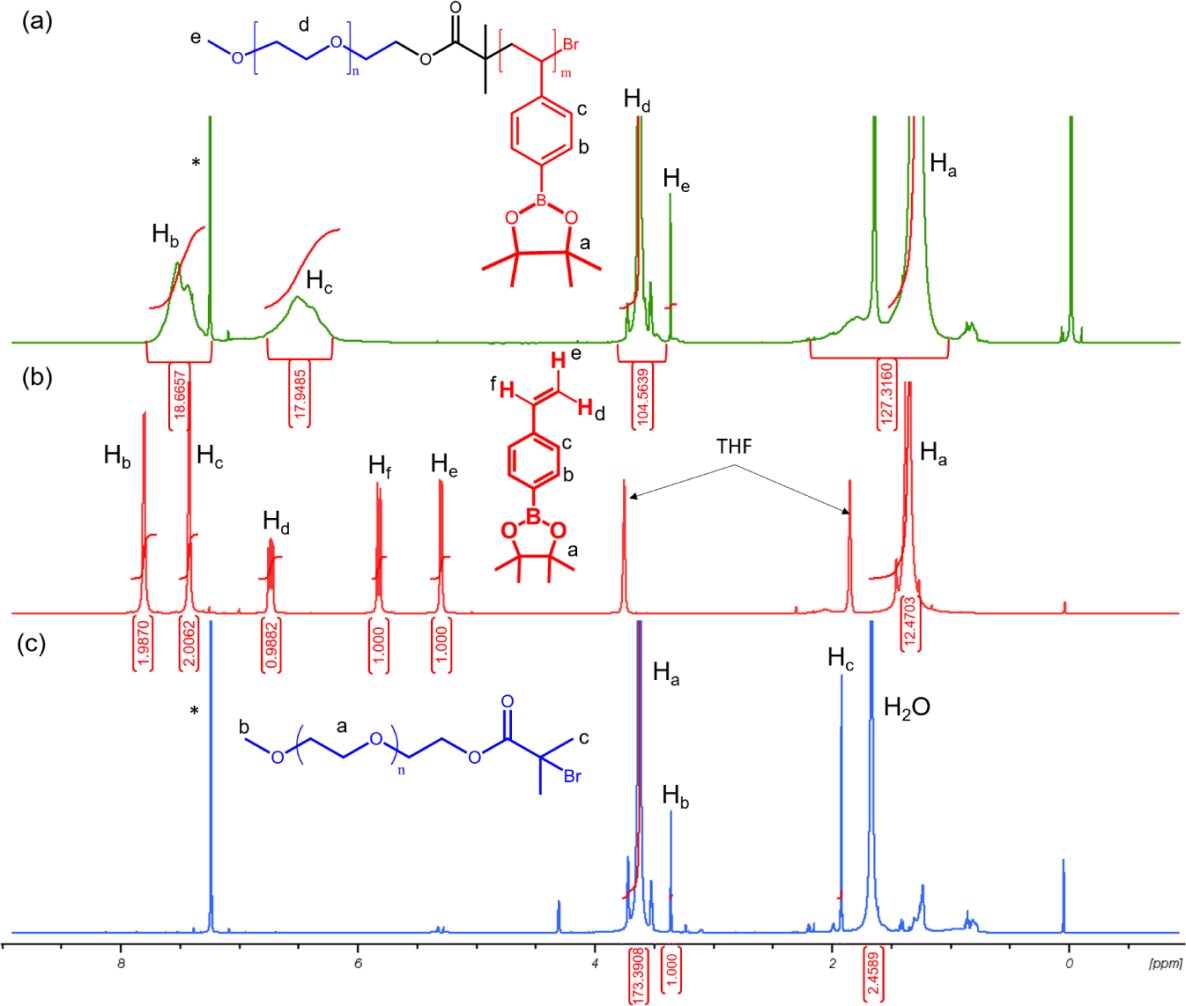
^1^H NMR spectrum of (a) mPEG-*b*-PBE_36_, (b) MBpin, and (c) mPEG-Br in CDCl_3_. * = CDCl_3_.

The synthesis of amphiphilic block copolymers with poly(ethylene glycol) (PEG) segments was achieved via chain extension from PEG-based atom transfer radical polymerization (ATRP) macroinitiators. The initial step of the block copolymer synthesis (Scheme 1) involved an esterification reaction between poly(ethylene glycol) methyl ether with a terminal hydroxyl group (PEG–OH) and 2-bromoisobutyryl bromide. The process yielded a bromide-end-functionalized PEG macroinitiator, denoted as mPEG-Br.^74^ The successful synthesis of mPEG-Br was confirmed through proton nuclear magnetic resonance (^1^H NMR) spectroscopy. As shown in Figure 1, a broad peak corresponding to the PEG backbone emerged within the range of 3.5–3.8 ppm (H_a_, −OCH_2_CH_2_−), and a distinct peak at 3.4 ppm, corresponding to the methoxy end group (H_b_, −OCH_3_). Additionally, resonances at 1.9 ppm (H_c_, −CBr(CH_3_)_2_) were observed, indicating the presence of a bromine atom. Crucially, the integration of peak c (1.9 ppm, −CBr(CH_3_)_2_) and peak b (3.4 ppm, −OCH_3_) demonstrated a ratio of 2.06:1. This ratio is close to the theoretical value of 2:1, thus confirming the retention of the bromine functionality during the esterification reactions. Moreover, the efficient of the esterification reaction for the mPEG-Br macroinitiator was determined by evaluating the integration ratio of H_c_/H_a_ using eq 3, as follows:
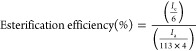
3where *I*_a_ and *I*_c_ represents the integrated area values of the peaks at 3.46–3.8 ppm (H_a_, −OCH_2_CH_2_−) and 1.9 ppm (H_c_, −CBr(CH_3_)_2_), respectively. The reaction conversion was determined to be 92%.^75^ To prevent catalyst deactivation during ATRP between 4-vinylphenylboronic acid and the copper catalyst, a preemptive measure was taken by protecting 4-vinylphenylboronic acid using pinacol following established literature procedures.^67^ The successful synthesis of the 4-vinylphenylboronic acid pinacol esters (MBpin) was validated by ^1^H NMR analysis. A broad signal at 1.2 ppm attributable to the pinacol group (H_a_, Ar–BO_2_(C(CH_3_)_2_)_2_) confirmed the successful protection of 4-vinylboronic acid (Figure 1b).

The chain extension of the MBpin chain from mPEG-Br was conducted through ATRP, utilizing CuBr as a catalyst and PMDETA as the ligand. This process was carried out in an oil bath maintained at 100 °C. The ^1^H NMR spectrum of mPEG-*b*-PBE_36_ (with a degree of polymerization (DP) of 36), presented in Figure 1a, revealed several distinct signals. Specifically, resonances at 7.2–7.6 ppm (H_b_, Ar–H) and 6–6.8 ppm (H_c_, Ar–H) were signals indicative of the successful polymerization of the boronic ester hydrophobic block. Additional proton resonances in the range of 0.9–1.5 ppm (H_a_, Ar-BO_2_(C(CH_3_)_2_)_2_) corresponded to the protected pinacol moiety, while the signal at 3.4 ppm (H_e_, -OCH_3_) was associated with the terminal methoxy protons. The degree of polymerization (DP) was determined utilizing an end-group analysis method, described by eq 4:^66^
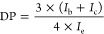
4where *I*_b_, *I*_c_, and *I*_e_ represent the integrated area values for signals at δ = 7.2–7.6, 6–6.8, and 3.4 ppm, respectively.

Variation in the lengths of the hydrophobic segments within the mPEG-*b*-PBE_36_ amphiphilic block copolymers were facilitated by adjusting the monomer-to-macroinitiator ratio and the duration of the, as detailed in Table S1. In our study, the primary factor influencing the regulation of the DP is the [*M*]:[*I*] ratio, which is also consistent with the mechanism of ATRP.^52^

**Figure 2:**
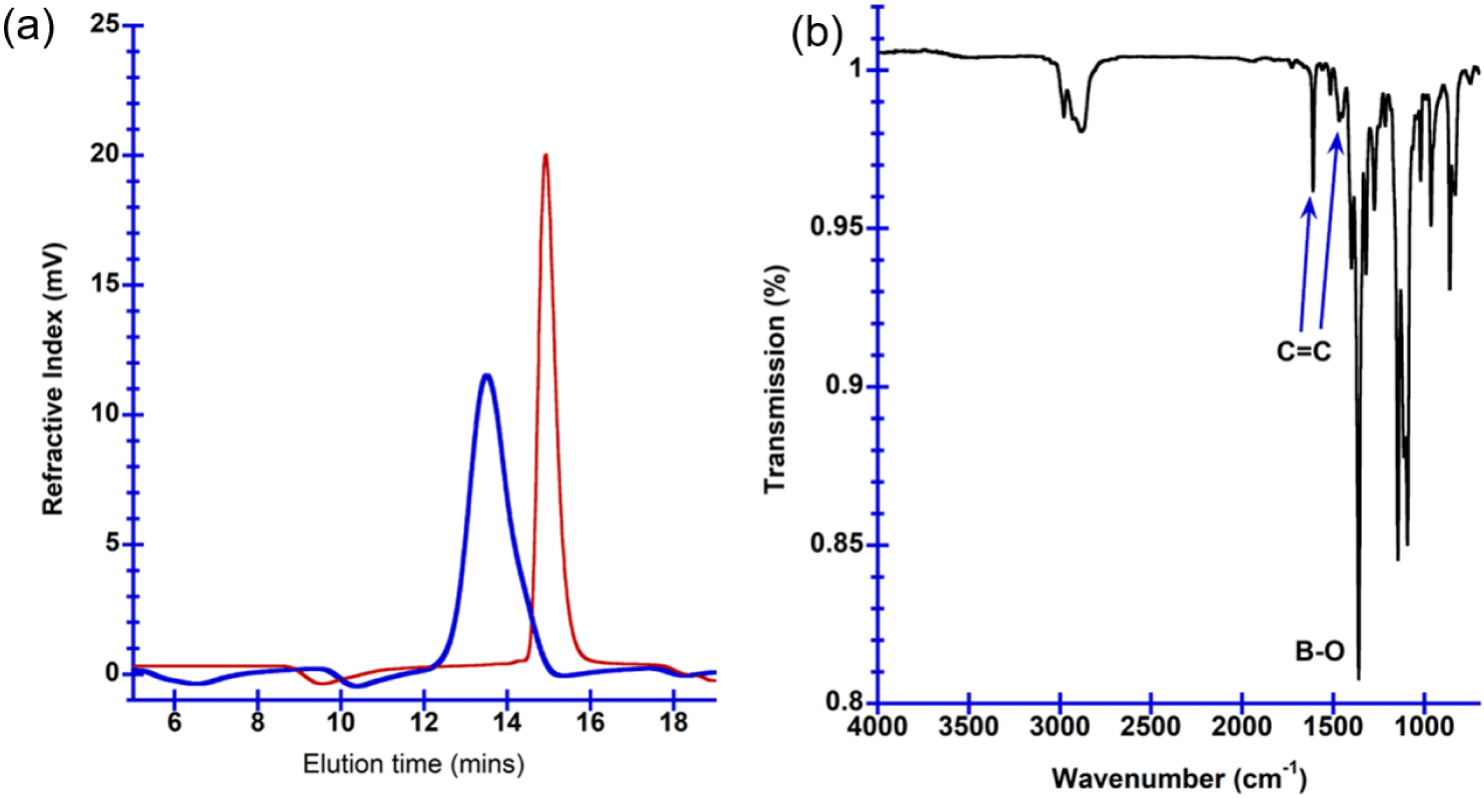
(a) GPC trace of macroinitiator mPEG-Br (red trace, *M*_n_ = 7402 g/mol, *M*_w_/*M*_n_ = 1.02) and mPEG-*b*-PBE_36_ (blue trace, *M*_n_ = 17 072 g/mol, *M*_w_/*M*_n_ = 1.13) and (b) FT-IR spectrum of mPEG-*b*-PBE_36_.

Gel permeation chromatography (GPC) was employed to determine the molecular weights and polydispersity indices (PDIs) of mPEG-Br and the mPEG-*b*-PBE_36_ block copolymer, as depicted in Figure 2a. The GPC profile of the mPEG-*b*-PBE_36_ block copolymer exhibited a notable shift toward higher molecular weights and a narrower polydispersity (*M*_w_/*M*_n_ ≤ 1.13) following the chain extension reaction via ATRP. This shift along with the narrow PDI unambiguously indicates the successful and controlled chain extension of all the PEG macroinitiators with MBpin, affirming the efficacy of the ATRP process in achieving the targeted polymer structure.

Fourier-transform infrared (FT-IR) spectroscopy analysis provides compelling evidence supporting the chemical structure of mPEG-*b*-PBE_36_, as previously indicated by ^1^H NMR findings. The FTIR spectra displayed distinct stretching vibration bands for the benzene C=C bond at 1609.9 and 1466 cm^–1^, and an intense stretching absorbance band from B–O bonding at 1350 cm^–1^, thereby confirmed the incorporation of the PBE block within the polymer backbone of mPEG-*b*-PBE_36_.^76^

### Self-Assembly of mPEG-*b*-PBE_36_ Micelles

**Figure 3:**
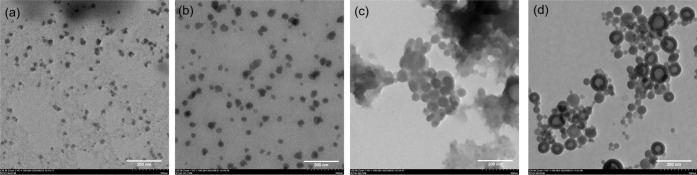
TEM size and morphology of the mPEG-*b*-PBE_*x*_ micelles prepared by Method 1 by using mPEG-*b*-PBE_*n*_ with varying DP of the PBE segment. (a) DP = 27, (b) DP = 36, (c) DP = 78, and (d) DP = 103. The average TEM sizes of the micelle aggregates are (a) 21 ± 4, (b) 31 ± 6, (c) 48 ± 3.8, and (d) 77 ± 15 (vesicles) and 42 ± 10 nm for the spherical micelle aggregates. The average size of the polymer micelles was calculated based on *n* = 30 particles.

The investigation into the size distribution and morphology of copolymer micelles was conducted by using dynamic light scattering (DLS) and transmission electron microscopy (TEM). DLS measurement provided insights into the size variations of mPEG-*b*-PBE_36_ micelles with differing DP values, as summarized in Table 1. A reduction in the ratio of the repeating units of the PEG corona to the PBE core correlates with an increase in the overall micelle size, as shown in Table 1 and Figure 3. Specifically, the size of the polymer micelles increased from 21 ± 4 nm for mPEG-*b*-PBE with a DP_corona/core_ of 4.18 to 48 ± 3.8 nm for mPEG-*b*-PBE with a DP_corona/core_ of 1.45. With a further reduction in the DP_corona/core_ ratio, leading to an increased proportion of the PBE segment, the micelles transitioned to a vesicular morphology, as depicted in Figure 3d. It is important to note that the observed sizes exceed those typically of unimolecular micelles, which form through the segregation of the hydrophobic block into the core and the hydrophilic blocks into the corona.^77,78^ Moreover, the micellar core size depicted in Figure 3 is larger than the estimated core size equation of *R*_core_ ∼ *N*_PBE_^0.4^*N*_PEG_^–0.15^, derived from Eisenberg’s early work.^78,79^

**Table 1:** Hydrodynamic Size of the mPEG-b-PBE_36_ Micelles Determined by DLS

block copolymer	DP_corona/core_^a^	*d*^b^(nm)	PDI^c^	*d*^d^(nm)	PDI^e^
mPEG-*b*-PBE_27_	4.18	40	0.227	70	0.273
mPEG-*b*-PBE_36_	3.14	68.5	0.148	152.6	0.186
mPEG-*b*-PBE_78_	1.45	92	0.175	215	0.177
mPEG-*b*-PBE_103_	1.09	115	0.152	242	0.132

a DP of the PBE_core_ block determined from ^1^H NMR and eq 4; DP of PEG_corona_ is fixed at 113.

b Number-average dimension of the copolymer micelles measured by DLS.

c Polydispersity of micelles measured by DLS.

d Number-average dimension of the lyophilized copolymer micelles measured by DLS.

e Polydispersity of lyophilized micelles measured by DLS.

While the literature frequently employs the term “polymer micelles” to denote various micellar structures emerging from the self-assembly of amphiphilic block copolymers, the stability of such large structures deviates from traditional unimolecular micelle mechanisms. The pioneering work of Eisenberg^77,79^ and others has elucidated the formation of large micelles with diameter over 100 nm, using various amphiphilic copolymers.^80,81^ Our results indicate that the structures observed in this study are not simple unimolecular micelles but rather multimolecular micelle with diameter ranging from 30 to 100 nm.^80^ Such multimolecular micelles, typically larger than 30 nm, are commonly observed in a polymer self-assembly system. The self-assembly of multimolecular micelles can be explained by the multimicelle aggregate (MMA) mechanism.^80,82^ The strong incompatibility between the PEG and PBE segments leads to microphase separation of the amphiphilic block copolymer into cone-shaped structures, which then aggregate into small micelle aggregates (SMAs) ranging from 20–50 nm in size, as shown in Figure 3a,b. As the hydrophobic PBE segment’s increase or the DP_corona/core_ decreases (Table 1), larger multimolecular micelle aggregate (MMA) form from the aggregation of SMA through intermolecular interactions such as hydrogen bonding. When the DP_corona/core_ is reduced to 1.09 (Table 1), the micellar morphology transitions to vesicular morphology as observed in crew-cut micelles.^83^

It is well documented that polymer micelles within the size range of 30 to 100 nm have been shown to accumulate effectively in highly permeable tumors.^84^ However, for tumors with poor permeability, micelles around 30 nm in size are preferred due to their enhanced tumor extravasation compared to their larger counterparts.^85^ Although the mPEG-*b*-PBE_27_ polymer system produces smaller-sized micelles, these micelles revealed a broad size distribution (PDI > 0.2, Table 1), which can adversely affect their biodistribution and drug delivery efficacy. Additionally, increasing the length of the hydrophobic segment (PBE) has been identified as a strategy to enhance the loading of boron-10, which is a critical factor in BNCT.^6^ Therefore, we have chosen to further optimize the mPEG-*b*-PBE_36_ polymer system, as it not only falls within the optimal size range for nanoparticle-mediated tumor targeting but also allows for increased ^10^B loading due to its larger hydrophobic PBE segment.

Herein, we explored the preparation of polymer micelles at concentrations near and exceeding the critical micelle concentration (CMC), respectively. It has been reported that at concentrations around the CMC, the polymer micelles tend to form loosely packed micellar aggregates.^86^ As the concentration of the block copolymer increases, the formation of denser micellar aggregates becomes possible.^87^ In this study, we investigated two self-assembly methods with differing initial mPEG-*b*-PBE_36_ block copolymer concentrations. Method 1 utilized an initial polymer concentration of approximately 0.6 mg/mL, significantly lower by a factor of 50 compared to that of Method 2. The resulting polymer micelles were analyzed by TEM and DLS (Figure 3). This finding confirms that the block copolymer concentration of 0.6 mg/mL surpasses the CMC for micelle formation. However, the resulting polymer micelles from Method 1 resulted in loosely packed aggregates and ill-defined morphology.^87^

**Figure 4:**
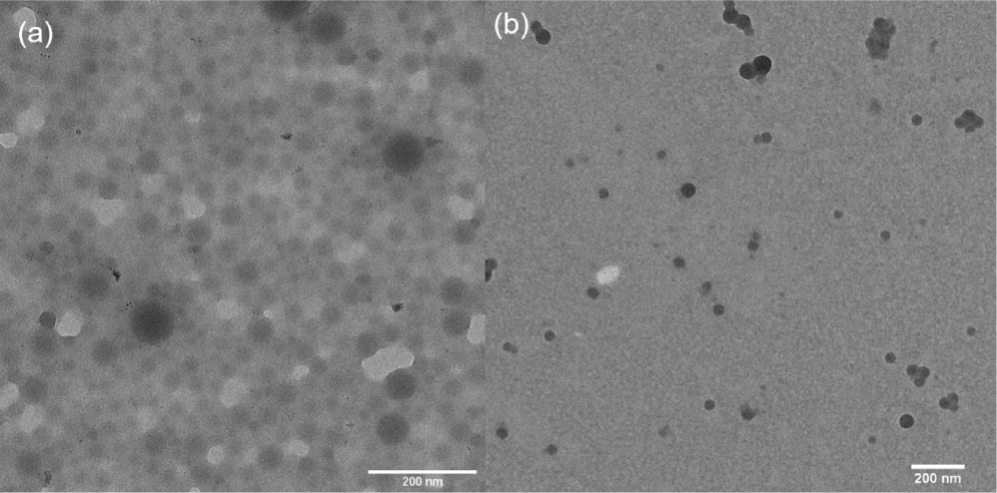
TEM morphology of (a) mPEG-*b*-PBE_36_ micelles synthesized by Method 2 (67 ± 17 nm, *n* = 20) and reconstituted using a probe sonicator for 30 s (43 ± 10 nm, *n* = 20) after lyophilization.

Method 2 incorporated a higher initial polymer concentration and the use of probe sonication during the self assembly to improve polymer dispersion.^86^ DLS analysis of mPEG-*b*-PBE_36_ micelles synthesized via Method 2 revealed an average size of approximately 67 nm, closely aligning with the 64 nm average hydrodynamic size obtained through Method 1. TEM imaging further elucidated the solid-state morphology of these micelles, demonstrating a spherical morphology with an average diameter of 67 ± 17 nm (*n* = 20) prelyophilization (Figure 4a) and 43 ± 10 nm (*n* = 20) postlyophilization (Figure 4b). Notably, micelles prepared using Method 2 were slightly larger than those produced by Method 1, an outcome attributed to the higher initial polymer concentration in Method 2.^86^ It should also be noted that micelle dimensions observed under the TEM are generally smaller than the hydrodynamic sizes measured by DLS in solution. This difference is due to TEM analyses being conducted on dried micelles, whereas DLS accesses the hydrodynamic diameter of polymer micelles in solution.^88^

### Lyophilization and Reconstitution of the Polymer Micelles

The stability of polymer micelles in aqueous media is compromised over time due to their propensity to aggregate and swell in solution,^58^ further exacerbated by the risk of microbial contamination during extended storage.^59^ Thus, lyophilization emerges as a practical and economical strategy to mitigate these challenges, although it introduces potential structural compromise of the micelles due to stress experience during the process.^61^ Consequently, the critical aspect of lyophilization pertains to the effective reconstitution of micelles. Herein, we investigated two reconstitution techniques: bath sonication and probe sonication, focusing on their efficacy in the reconstitution of mPEG-*b*-PBE_36_ micelles.

Ultrasound technology, commonly employed for reconstituting lyophilized micelles,^89^ facilitates particle dispersion in solutions. Bath sonicators, optimal for large-volume and dilute lipid dispersion, contrast with probe sonicators, which are preferred for small-volume dispersions due to their with higher energy input.^90^ Notably, cavitation – central to ultrasound’s effectiveness – occurs unevenly at the bottom of the water bath in bath sonicators, whereas probe sonicators induce cavitation directly within the sample container, ensuring uniformity and reproducibility.^91^ This makes probe sonication favorable for the reconstitution of lyophilized mPEG-*b*-PBE_36_ micelles.

**Figure 5:**
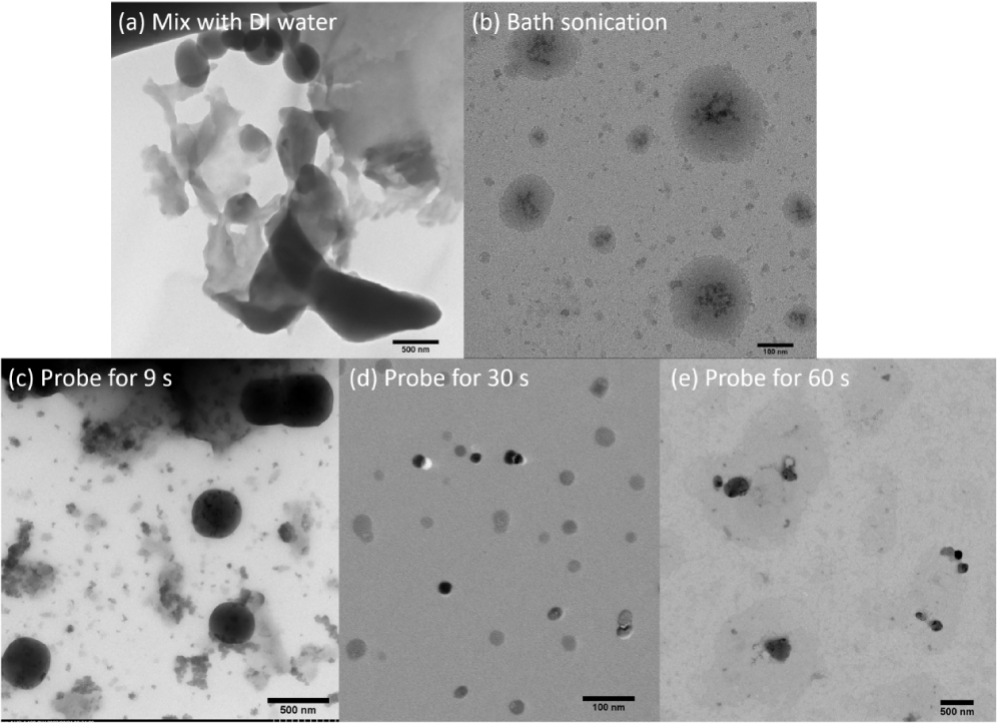
TEM morphology of the lyophilized mPEG-*b*-PBE_36_ micelles synthesized by Method 1 and reconstituted by (a) mixing with DI water only and (b) using an ultrasonic bath sonicator. The lyophilized mPEG-*b*-PBE_36_ micelles synthesized by Method 1 and reconstituted by using the probe sonicator for (c) for 9 s, (d) for 30 s, and (e) for 60 s.

Our study methodically evaluated the impact of ultrasonic treatment using both sonication techniques across varying durations, from 9 to 60 s, while keeping the concentration of the reconstituted polymer micelles consistent. TEM analysis showed that the lyophilized polymer micelles reconstituted by simple dilution with deionized (DI) water exhibited significant aggregation and lacked the uniformity required for drug delivery purposes (Figure 5a). Only a few multimicelle aggregates (MMAs) were evident after lyophilization and reconstitution in DI water. In contrast, micelles reconstituted using bath sonication revealed the self-assembly of smaller SMA and unimolecular micelles, which appeared as darker contrast structures within the cores of larger multimicelle aggregate (MMA) (Figure 5b).^80,81^ Additionally, TEM analysis shown in Figure 5b indicated the presence of two distinct micelle populations: larger MMAs with an average size of 144 ± 75 nm and smaller SMA measuring approximately 22.6 ± 4 nm upon reconstitution using bath sonication. The formation of such large and polydispersed MMAs can be attributed to the freezing process during lyophilization, which likely introduced various phases and interfaces that disrupted the structure and stability of the polymer micelles. Upon reconstitution in the bath sonicator, most polymer micelles reassembled into smaller SMAs with an ill-defined morphology. However, some polymer micelles remained largely aggregated as MMAs. This larger, nonuniform aggregation of MMAs can be linked to significant variation in cavitation effects at the base of the water bath during sonication, leading to the formation of polydispersed micellar structures.^90^

Next, we evaluated the impact of probe sonication, operated at a constant power and frequency (125 W, 20 kHz), on the size and morphology of the polymer micelles over varying sonication durations: 9, 30, and 60 s. Initial observations at 9 s of probe sonication revealed that only a subset of the micellar population attained a spherical morphology, while the majority of micelles displaying aggregation (Figure 5c). When the probe sonication duration was increased to 30 s, a significant transformation was noted; the majority of micelles achieved a uniform size and size distribution (25 ± 4 nm, *n* = 20) and exhibited a spherical morphology (Figure 5b). The aggregated micelle populations that was observed in the 9-s samples disappeared, with simultaneous increase in the concentration of the dispersed, uniform sized spherical micelles. However, further extending the sonication time to 60 s led to the degradation of the micellar structure (Figure 5c). This structural compromise is likely attributable to the overheating effects associated with prolonged sonication time.^92^ These findings underscore the critical balance required in the application of probe sonication for the reconstitution of polymer micelles after lyophilization. While a moderate duration of sonication (30 s) promotes uniform dispersion and ideal micellar morphology, excessive sonication (60 s) poses a risk to structural integrity due to thermal stress.

### Stability of the mPEG-*b*-PBE_36_ Polymer Micelles

**Figure 6:**
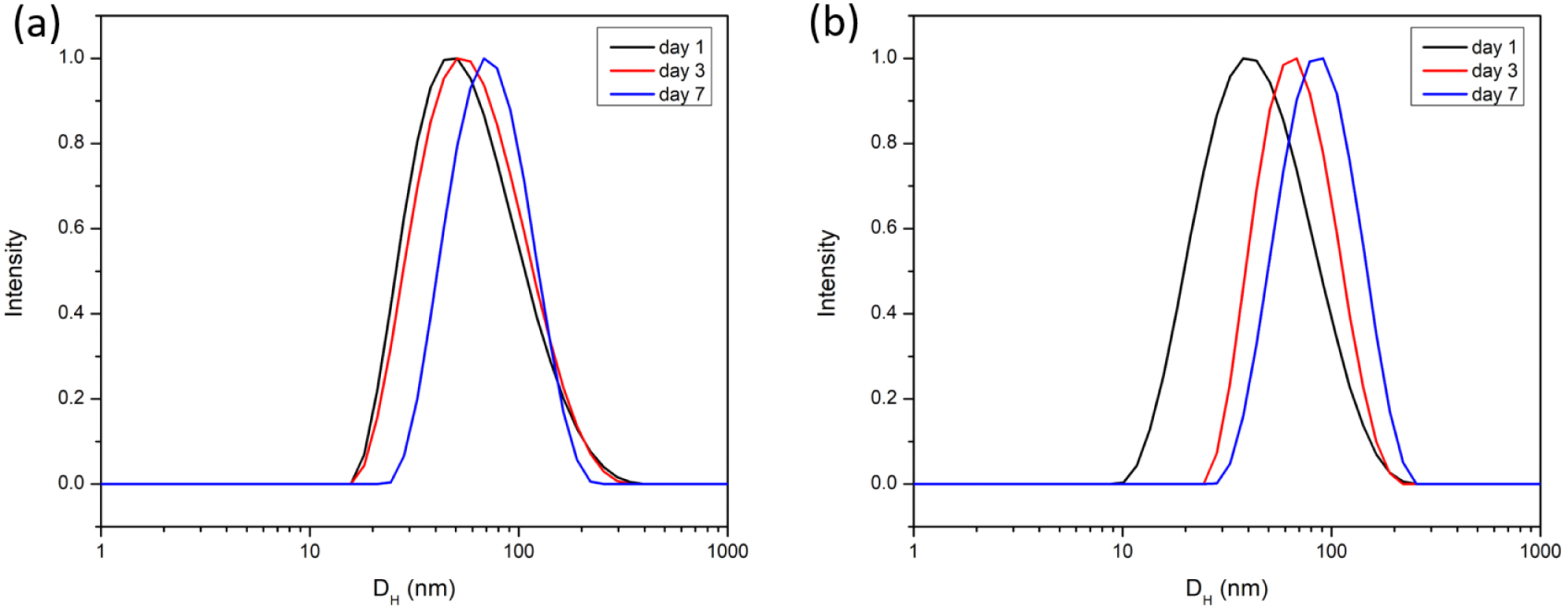
Stability of mPEG-*b*-PBE_36_ micelles incubated in (a) PBS and (b) DMEM over 7 days. The hydrodynamic size of mPEG-*b*-PBE_36_ was determined by DLS.

To evaluate the long-term stability of mPEG-*b*-PBE_36_ micelles, critical for their application in forthcoming in vivo experiments, stability assessments were conducted in phosphate-buffered saline (PBS) and Dulbecco’s modified Eagle medium (DMEM).^93^ DLS was utilized to monitor the size and size distribution of freshly synthesized micelles in PBS and DMEM from day 1 to day 7 as documented in Figure 6. The mPEG-*b*-PBE_36_ micelles displayed remarkable stability when incubated in PBS over a 7-day period, with no observable swelling or degradation, maintaining a stable hydrodynamic size of approximately 67 nm. This indicates that mPEG-*b*-PBE_36_ micelles possess excellent stability characteristics, suitable for further in vitro and in vivo experiments. Upon incubation in DMEM supplemented with 10% FBS, the mPEG-*b*-PBE micelles demonstrated a significant increase in size over time, with their hydrodynamic diameter expanding from 51.4 to 99.9 nm. This observed increased in the hydrodynamic size may be attributed to the formation of a stable hard protein corona around the micelles, a phenomenon commonly encountered with nanoparticles in complex biological environments.^94,95^ While the formation of protein corona on nanoparticle drug carriers in vivo is inevitable, it is crucial to ensure that the resulting equilibrium size of the polymer micelles remains within the optimal range for effective blood circulation and tumor accumulation. The size range observed in this study remains conducive to exploit the enhanced permeation and retention (EPR) effect, a key mechanism facilitating the accumulation of therapeutic agents in tumor tissues due to their leaky vasculature and impaired lymphatic drainage.^96,97^ Despite the potential challenges posed by serum protein interactions, the mPEG-*b*-PBE_36_ micelles maintained a size range that supported their application in tumor targeting and drug delivery, leveraging the EPR effect for improved therapeutic outcomes.

### Cytotoxicity Evaluation of mPEG-*b*-PBE Micelles

**Figure 7:**
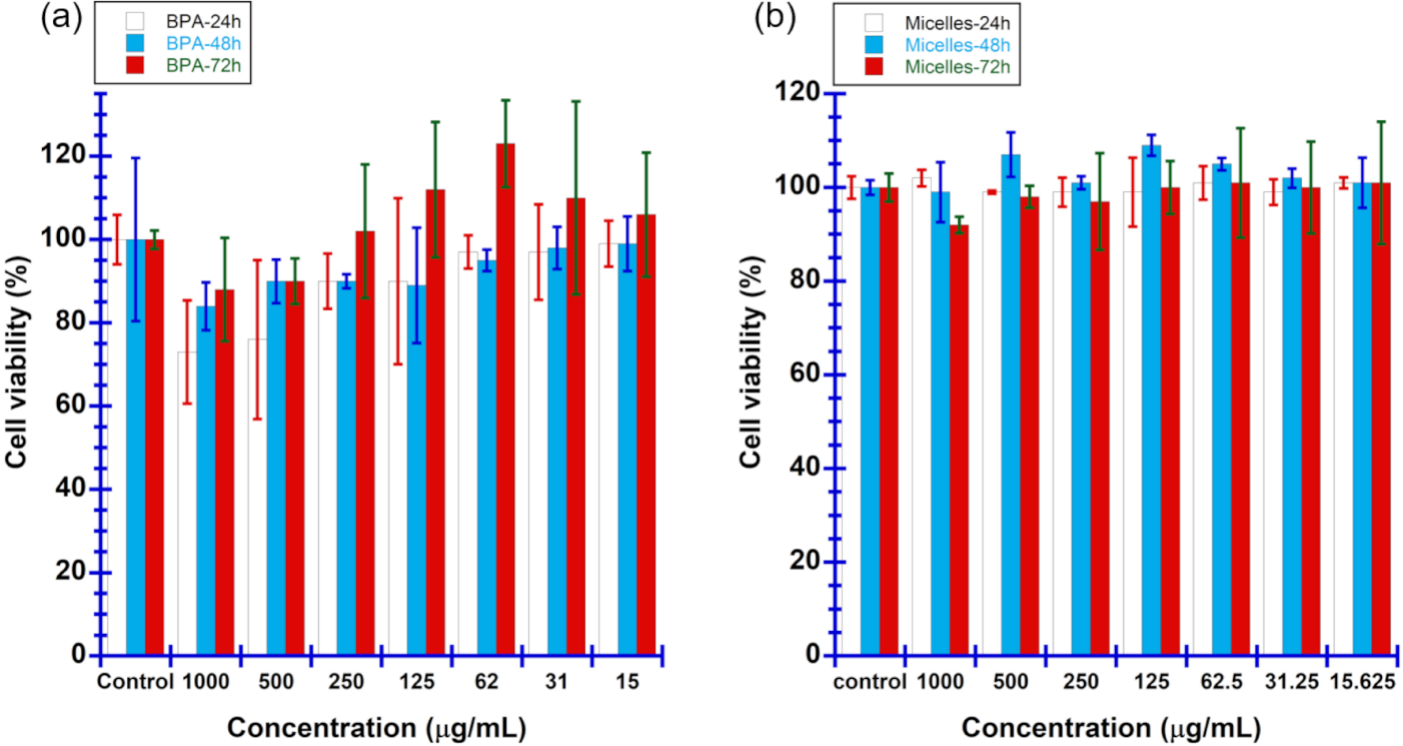
Time-dependent cytotoxicity of (a) the mPEG-*b*-PBE_36_ micelles and (b) BPA was evaluated by using the MTS assay. The results are expressed in means ± SD (*n* = 3 for all experimental groups). *p < 0.5 versus control (0 μg/mL concentration), **p < 0.05 versus control (0 μg/mL concentration), ***p < 0.005 versus control (0 μg/mL concentration).

The cytotoxicity of mPEG-*b*-PBE_36_ micelles, in comparison to BPA, was accessed by exposing B16F10 melanoma cells to various concentrations of these boron-containing drugs for periods of 24, 48, and 72 h. The objective was to evaluate their potential efficacy and safety as boron carriers for BNCT. As depicted in Figure 7a, mPEG-*b*-PBE_36_ micelles exhibited exceptional biocompatibility, with cell viability remaining above 92% across all tested concentrations, up to 1000 μg/mL. This high level of biocompatibility emphasizes the potential of mPEG-*b*-PBE_36_ micelles as promising boron nanodrugs in BNCT applications, highlighting their safety profile even at high concentrations.^98^ Conversely, the BPA-treated group demonstrated a significant induction of cell apoptosis, exceeding 25% cell at a concentration of 1000 μg/mL after 24 h of incubation. Notably, cell viability in the BPA group improved to around 90% after 72 h, as illustrated in Figure 7b.^99^ This improvement in cell viability over time suggests a possible elimination of BPA from the cellular environment, which may reduce its potential for sustained cytotoxic effects during BNCT. Our in vitro cytotoxicity studies indicate that the mPEG-*b*-PBE_36_ micelles exhibit reduced cytotoxicity in relation to BPA, an FDA-approved boron drug for BNCT. This study underscores not only the micelles’ suitability as boron drug candidates for BNCT but also their potential advantage in terms of reduced cytotoxicity and enhanced biocompatibility in cancer treatment.

### Cellular Uptake Efficiency of mPEG-*b*-PBE_36_ Micelles

**Figure 8:**
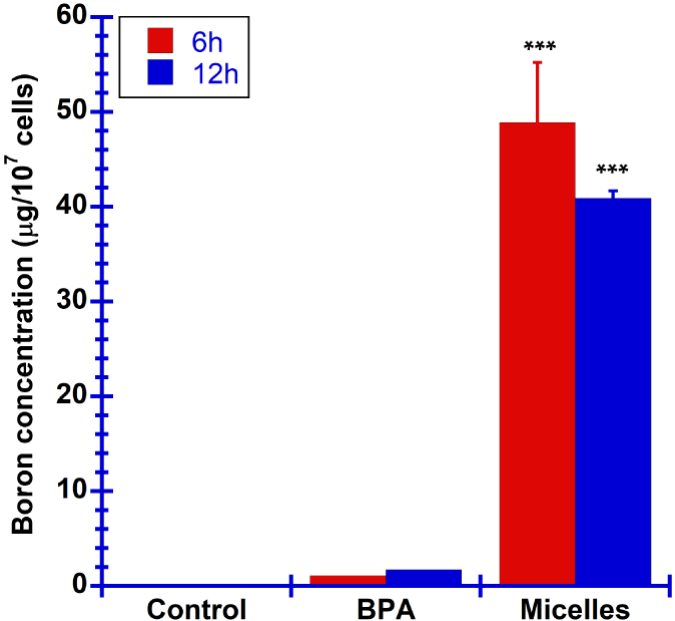
ICP-MS analysis of the boron contents internalized by the cell at 6 and 12 h of incubation times. The results are expressed in means ± SD (*n* = 2 for all experimental groups). *p < 0.5 versus BPA, **p < 0.05 versus BPA, ***p < 0.005 versus BPA.

The cellular uptake of mPEG-*b*-PBE_36_ micelles by B16F10 melanoma cells was investigated by using ICP-MS analysis and confocal imaging. Cells were treated with mPEG-*b*-PBE_36_ micelles and BPA at a concentration of 1 mg/mL, respectively. As illustrated in Figure 8, the copolymer micelles achieved significantly higher boron accumulation within cells at both 6 and 12 h incubation time periods compared to BPA. Specifically, a 38-fold increase in boron accumulation was observed at the 6 h mark for mPEG-*b*-PBE_36_ micelles in relation to BPA, with this ratio reducing slightly to 28-fold after 12 h. This marked increase in cellular uptake of mPEG-*b*-PBE_36_ micelles highlights their superior efficacy as a delivery vehicle for boron in the context of BNCT. The enhanced cellular uptake of mPEG-*b*-PBE_36_ micelles is likely due to their optimal equilibrium hydrodynamic size of 99.9 nm in biological media, which facilitate efficient internalization and accumulation within cancer cells by exploiting the EPR effect.^96^ Moreover, the modification of the micelles with polyethylene glycol (PEG) enhances their biocompatibility and prolongs their circulation time in the bloodstream, making them ideal nanocarriers for delivering the ^10^B isotope necessary for effective BNCT.^100^ For effective BNCT treatment, an accumulation of 10^9^ boron-10 atoms per cell is necessary to induce cancer cell death.^4^ Given the presence of naturally occurring ^10^B isotope at approximately 20% and the substantial boron atom count per polymer micelles, mPEG-*b*-PBE_36_ micelles reached an approximate accumulation of 10^17 10^B atoms per cell following a 6 h incubation period. In contrast, BPA achieves an accumulation of approximately 10^16 10^B atoms per cell. This significant boron accumulation by mPEG-*b*-PBE_36_ micelles, therefore, affirms their enhanced capability for targeted boron delivery for effective treatment of cancer via BNCT.

### Encapsulation of Coumarin 6 within the mPEG-*b*-PBE_36_ Micelles

Polymer micelles, due to their unique amphiphilic structure, have emerged as pivotal carriers and solubilizers in drug delivery systems, demonstrating significant clinical relevance.^101^ The hydrophilic corona of these micelles is adept at evading opsonization, while their capacity to exploit the enhanced permeability and retention (EPR) effect enables them to achieve favorable biodistribution and specific tumor targeting.^96^ The structural design of polymer micelles also allow for the incorporation of highly toxic or poorly soluble small-molecule drugs and imaging contrast agent through chemical conjugation or physical entrapment, effectively delivering therapeutic agents to diseased sites and minimizing exposure to healthy tissues.^55,102^

**Figure 9:**
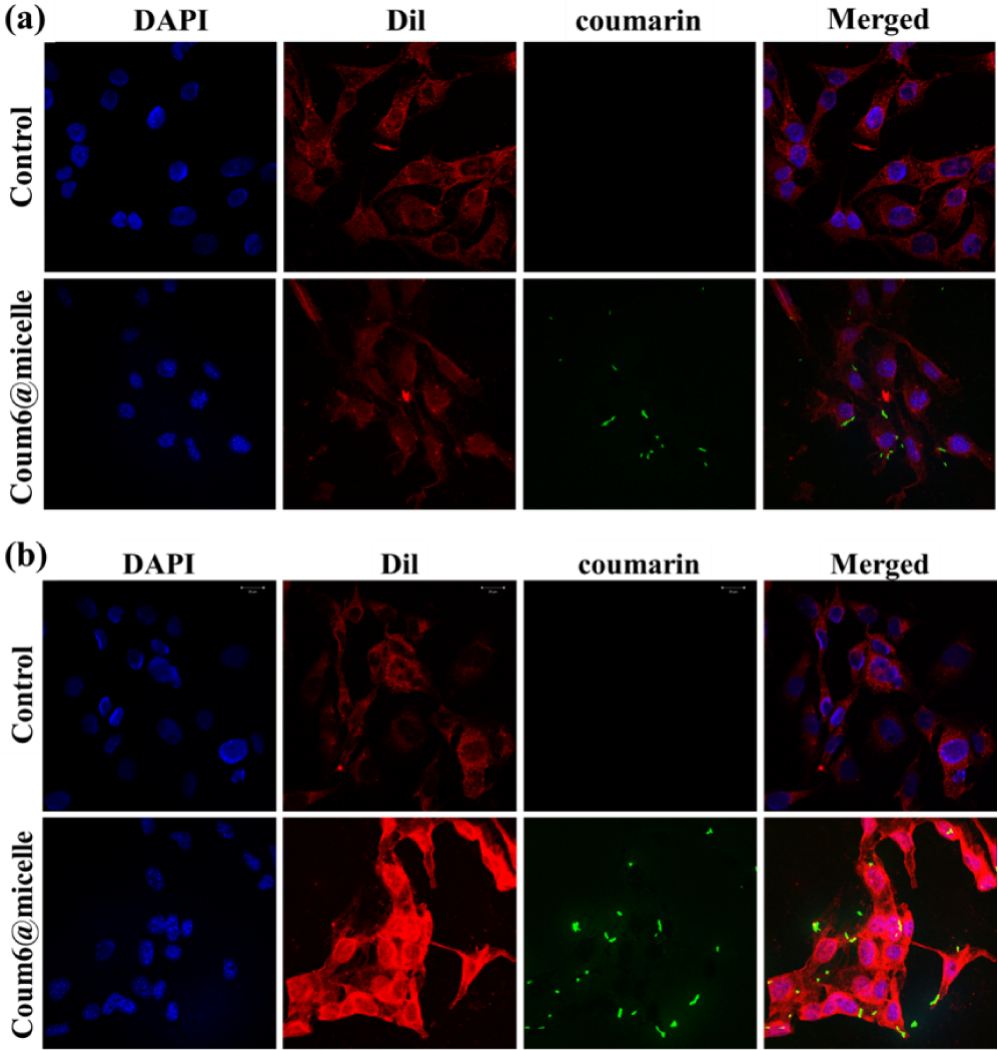
Confocal images of mPEG-*b*-PBE_36_ micelle uptake for (a) 6 h and (b) 12 h by B16F10 melanoma cells. In both images, the cell nuclei are stained with DAPI (blue fluorescence), indicating the location of the nuclei. The cell membranes are stained with DiI staining (red fluorescence), highlighting the cellular boundaries. The fluorescence of the coumarin-loaded micelles (green) reveals the distribution of the micelles within the cells.

In this study, the hydrophobic core of the mPEG-*b*-PBE_36_ micelles was employed to encapsulate coumarin 6 (coum6), a compound known for its green fluorescence, to access cellular uptake in B16F10 melanoma cells via confocal microscopy. Figure 9 shows the internalization of coumarin 6-loaded mPEG-*b*-PBE_36_ micelles within B16F10 melanoma cells, evident from the pronounce green fluorescence emission. Notably, the fluorescence micelles dispersed throughout the cytoplasm and approached the vicinity of the cell nucleus, a strategic positioning that may enhance the efficacy of cell apoptosis induction via the α-particles and ^7^Li produced during BNCT therapy. Additionally, an increase in the fluorescence intensity from 6 to 12 h of incubation was observed, corroborating the findings from ICP-MS analysis regarding enhanced cellular uptake over time. In comparison, control samples without mPEG-*b*-PBE_36_ micelle incubation displayed only the inherent fluorescence signals from the nuclei and cytoplasm of the melanoma cells, lacking the green fluorescence indicative of coumarin 6.

### In Vitro BNCT Efficacy on B16–F10 Melanoma Cells with mPEG-*b*-PBE_36_ Micelles

**Figure 10:**
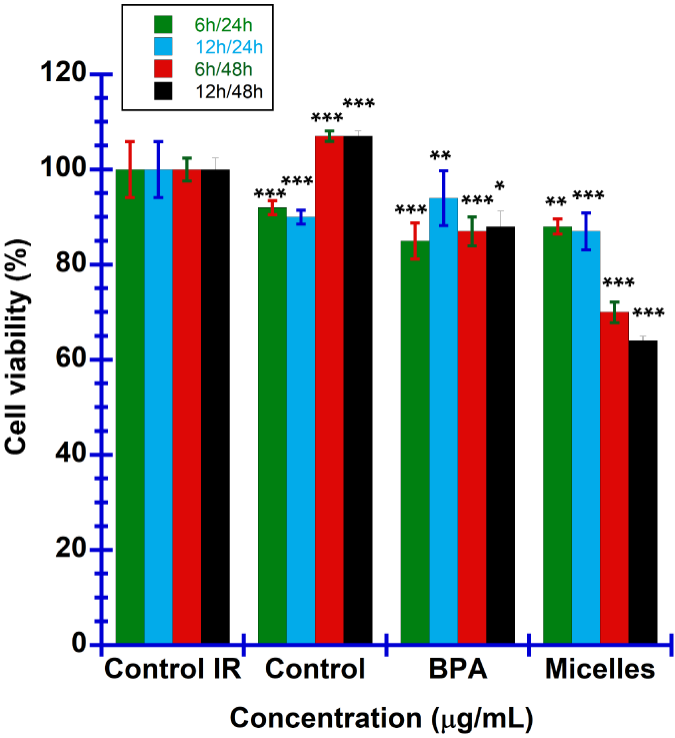
Survival rates of B16–F10 melanoma cells as a function of incubation times (6 and 12 h) and postirradiation intervals (24 and 48 h). Cells were treated with 1 mg/mL boron agents and subjected to 30 min of neutron irradiation. The data are presented as means ± SD (*n* = 4 for all experimental groups). Statistical significance is denoted as *p < 0.5, **p < 0.05, ***p < 0.005 compared to the control group.

The impact of BNCT on the survival of B16–F10 melanoma cells was accessed by comparing cell viability across different incubation durations with boron-containing drugs, followed by a postirradiation period. Figure 10 illustrates that the group subjected to irradiation experienced approximately 10% cell apoptosis, in contrast to the nonirradiated control group. This observation is likely due to the excessive generation of reactive oxygen species (ROS) during BNCT, leading to cellular damage.^10^ However, a remarkable recovery was noted in the melanoma cells 48 h postirradiation incubation, with survival rates even exceeding those of the nonirradiated controls, suggesting that neutron irradiation, in the absence of boron drug, does not cause fatal cellular damage. When analyzing the cell viability within the 24 h postirradiation period, it was observed that cells incubated with BPA for 6 h displayed a lower survival rate compared to those incubated for 12 h. This outcome may be linked to BPA’s peak intracellular accumulation occurring within 2 h of incubation, with its effectiveness decreasing over extended periods.^103^ In contrast, cells treated with 1 mg/mL mPEG-*b*-PBE_36_ micelles for both 6 and 12 h periods exhibited more than 10% cell apoptosis within 24 h postirradiation incubation. Notably, 48 h postirradiation, apoptosis rates exceeded 30% in both groups, with the 12 h incubation period showing lower cell viability in relation to the 6 h counterpart, due to the higher intracellular boron accumulation, as confirmed by ICP-MS analysis (Figure 8). These findings highlight the superior efficacy of mPEG-*b*-PBE_36_ micelles over BPA as a boron carrier for BNCT, as demonstrated by their enhanced in vitro cytotoxicity against melanoma cells.

### In Vivo Biodistribution of mPEG-*b*-PBE_36_ in a B16–F10 Melanoma Mouse Model

**Figure 11:**
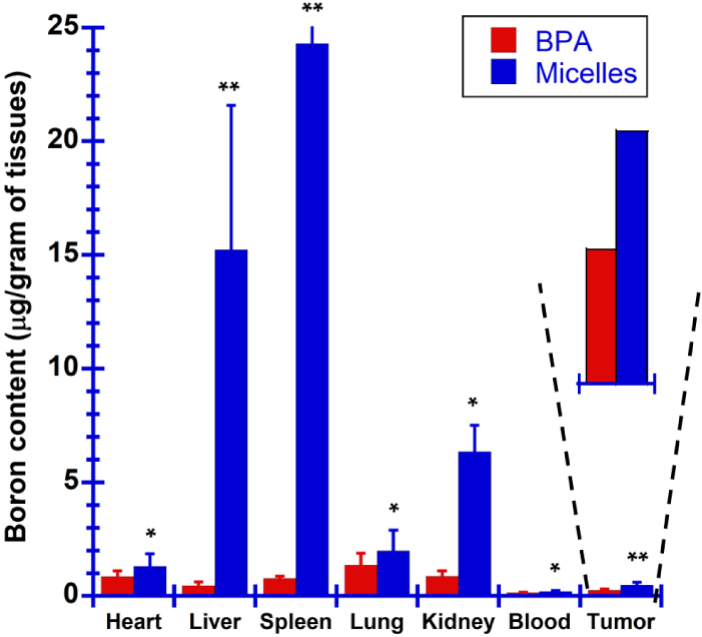
Boron biodistribution of the B16–F10 melanoma tumor-bearing mouse received intravenous (i.v.) injections of BPA and mPEG-*b*-PBE_36_ micelles at a concentration of 100 mg/kg every 2 days, totaling 3 injections. The results are expressed in means ± SD (*n* = 3 for all experimental groups). *p < 0.5 versus BPA, **p < 0.05 BPA.

In biodistribution of mPEG-*b*-PBE_36_ in an in vivo B16–F10 melanoma mouse model was examined to evaluate their potential efficacy in BNCT. Following the establishment of the melanoma model through subcutaneous injection of B16–F10 melanoma cells into the right hind limb. Seven days post-tumor inoculation, when the tumor volume reached approximately 100 mm^3^, both BPA and mPEG-*b*-PBE_36_ micelles were administered intravenously across three injections. By the time of the final injection, the average tumor size in all experimental groups had increased to about 250 mm^3^. 24 h after administering the last dose, the mice were euthanized and the organs and tumors were harvested for boron concentration analysis using ICP-MS. Significant accumulation of the micelles within the spleen and liver was observed, as illustrated in Figure 11. This phenomenon is likely a result of the intravenous administration route, whereby bloodborne proteins may induce aggregation of the mPEG-*b*-PBE_36_ micelles, increasing their size and facilitating macrophage-mediate clearance into the liver.^104^ Despite these challenges, tumor tissues from mice treated with the polymer micelles demonstrated an approximately 2-fold increase in boron concentration (0.47 μg of B/g of tissues) compared to that observed with the administration of the state-of-the-art BPA drug. However, this 2-fold increase in tumor accumulation via an EPR effect, while significant, remains lower than previously reported values in the literature.^24,25,85,100,105^ This discrepancy could be attributed to several factors including variations in cancer types,^106^ tumor vasculature, and the inherent characteristics of the tumor microenvironment,^106,107^ all of which are known to significantly influence the efficiency of EPR-based targeting.^108−110^ Moreover, the relatively large initial tumor size (>250 mm^3^) in all experimental groups may further explain the subdued enhancement in tumor accumulation observed.^111^ For instance, research indicates that smaller tumors tend to receive a higher radiation dose effectively compared to larger tumors when the same therapeutic agent is applied at identical dosages.^112−114^ Moreover, larger tumor sizes have been linked to poorer vascular networks, particularly in the tumor core, leading to inadequate delivery and distribution of therapeutic agents, including polymer micelles.^115^ Clinically, there is a well-documented correlation between tumor size and response rates to treatments, suggesting that larger tumors often respond less effectively to therapy due to factors such as internal necrosis and poor vascularization, which are detrimental to the efficient delivery of nanoparticle-based therapies.^116,117^ Nevertheless, the polymer micelles in this study displayed improved tumor targeting, achieving a T/B ratio of 2.5. In contrast, the BPA-treated group exhibited poor tumor selectivity with a T/B ratio of only 1.8. A high T/B ratio is imperative for the effective application of BNCT to minimize collateral damage to the normal tissues. The findings of this study underscore the potential of polymer micelles as a more efficacious delivery vehicle for boron in BNCT, offering both enhanced boron accumulation in tumor tissues and improved selectivity compared to traditional BPA drug formulations.

### In Vivo BNCT Efficacy in the B16–F10 Melanoma Mouse Model

**Figure 12:**
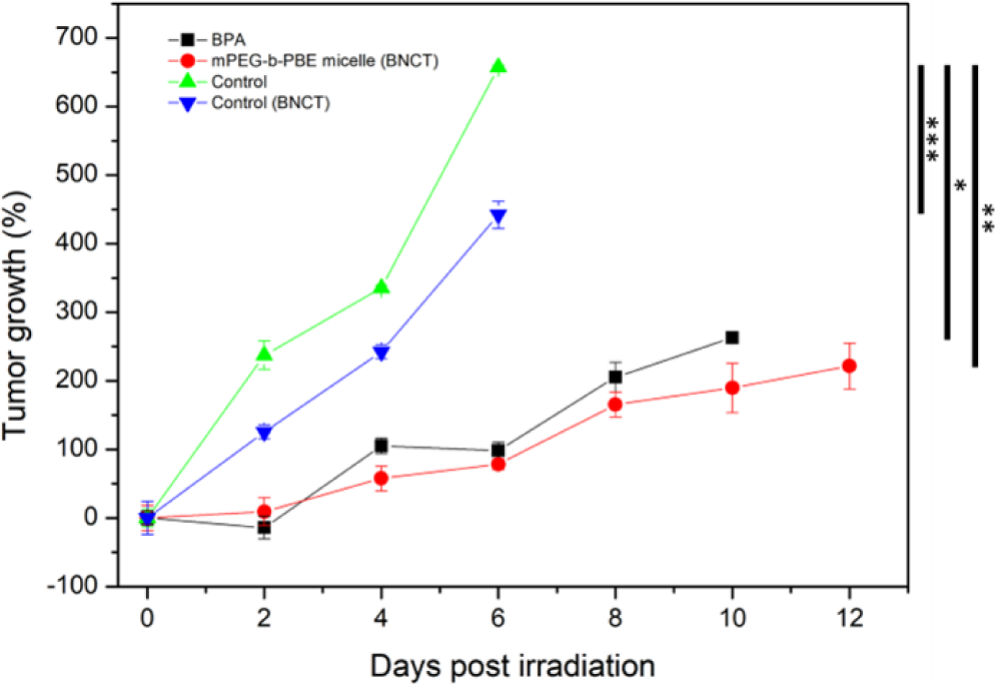
Tumor growth of B16F10 melanoma mouse after BNCT. The results are expressed in means ± SD (*n* = 3 for all experimental groups). *p < 0.5 versus control, **p < 0.05 versus control, ***p < 0.005 versus control.

The B16–F10 melanoma mice were divided into four groups at random, each comprising five mice (*n* = 5): (1) control group, (2) control group (BNCT), (3) BPA (BNCT), and (4) mPEG-*b*-PBE_36_ micelles (BNCT). The designation “BNCT” within parentheses indicates groups that received neutron irradiation, whereas its absence signifies groups that did not undergo irradiation. Over 12 days following BNCT treatment, tumor volumes were measured with calipers. The findings, illustrated in Figure 12, revealed that a rapid increase in tumor volume in the nonirradiated control group exhibited swift escalation, surpassing 1000 mm^3^ by day 7, necessitating euthanasia in accordance with IACUC guidelines. During the first 6 days, the irradiated control group showed a slight reduced tumor growth rate compared to the nonirradiated control group. Tumor doubling time (DT) for untreated and treated tumors was calculated, along with tumor growth delay (TGD), using the following equations:
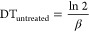
5
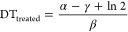
6
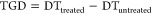
7where α represents the log tumor volume at the onset of the treatment, β is the relative rate of tumor growth, and γ is derived from the relationship between α and β (2e^α^= e^γ+β*t*^). The melanoma tumors’ rapid proliferation and the resulting poorly structured vascular system within solid tumors impede the efficient delivery of therapeutic agents to distal cells. Despite these limitations, the treated groups demonstrated significant tumor growth inhibition. Specifically, mice treated with BPA experienced a tumor growth delay of 3.38 days, while those treated with mPEG-*b*-PBE_36_ micelles showed a delay of 5 days (Table S3),^118^ along with sustained tumor growth inhibition until the 12th day postirradiation. Compared to mice in the BPA group, only one mouse survived the 10th day of treatment as shown in Figure 12 (black trace). Moreover, our study’s findings indicate that tumor inhibition efficacy in groups treated with BPA does not significantly differ from that observed in the control group, as evidenced by a *P*-value greater than 0.5. Conversely, the tumor inhibition observed in mice treated with mPEG-*b*-PBE micelles demonstrated a statistically significant difference when compared to the control group, with a *P*-value less than 0.05. Despite the promising attributes of the boron-rich polymer micelles as a potent BNCT drug, the therapeutic efficiency reported in this study showed only moderate enhancement compared to free drugs. This outcome is likely attributable to the dosage of boron-10 (B-10) used. In our study, the synthesis of polymer micelles utilized non-B10 enriched precursors. Consequently, the administration of 100 mg of micelles/kg mouse body weight translated to only 4.6 mg B10/kg mouse body weight, based on the 20% of naturally occurring B10 and the composition of the polyboronate ester in the mPEG-*b*-PBE micelles. In this context, previous in vivo BNCT studies typically administered B10 dosage ranging from 10 to 100 mg of ^10^B/kg mouse body weight.^24,25,100,105^ This discrepancy in B10 dosage is a plausible explanation for the moderate BNCT treatment efficacy observed in our study compared to previous reports. Our findings aligned with those of Nagasaki et al. who also employ nonisotope enriched phenylboronic acid-decorated nanoparticles for BNCT. In their study, the nanoparticle-based drug showed similar tumor inhibition effectiveness as the free drug.^47^ Additionally, the rapid growth of B16–F10 metastatic melanoma resulted in a large initial tumor size of 250 mm^3^ at the onset of the treatment. This is significantly larger than the initial tumor sizes of 50 to 100 mm^3^ typically reported in other in vivo studies reported in the literature.^24,25,85,100,105^ As previously discussed, the larger tumor size and the heterogeneity of the tumor microenvironment can severely restrict the extravasation and penetration of polymer micelles into the solid tumor.^108−110^ This limitation not only affects drug delivery but also results in poorer therapeutic outcomes. These findings suggest that both the lower B10 dosage and the challenges posed by larger tumor sizes and their microenvironmental complexities are critical barriers to achieving higher therapeutic efficacy in BNCT by using polymer micelles.

### Immunohistochemical Analysis of Tumor Tissue for BNCT Efficacy with mPEG-*b*-PBE_36_ Micelles

**Figure 13:**
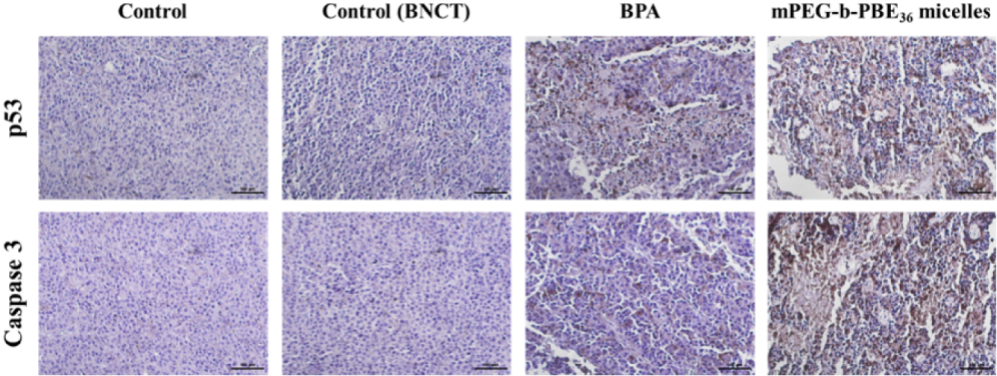
Immunohistochemical analysis of the p53 protein and caspase 3. Scale bar = 100 μm.

To elucidate the therapeutic efficacy of mPEG-*b*-PBE_36_ micelles in BNCT, we conducted immunohistochemical analyses on tumor tissue sections from mice treated with BPA and mPEG-*b*-PBE_36_ micelles, respectively. The analyses focused on staining for p53 and caspase-3, which are markers for cell migration^119^ and apoptosis,^120^ respectively. Our findings revealed negligible expression of p53 biomarkers in both the control and the BNCT-control groups. In contrast, tissue sections from mice treated with BPA and mPEG-*b*-PBE_36_ micelles displayed distinct brown coloration, indicative of an activated p53 pathway, suggesting enhanced cancer cell suppression due to the treatments (Figure 13). Notably, tissue sections from the mPEG-*b*-PBE_36_ micelle-treated group exhibited deeper brown staining, signifying more extensive tumor cell damage. Further analysis using caspase-3 antibody staining to access apoptosis levels post-BNCT treatment showed significant apoptosis in the mPEG-*b*-PBE_36_ micelle-treated group, as evidenced by pronounced brown staining. This starkly contrasts with the minimal apoptotic indicators in both control groups. These immunohistochemical findings strongly suggest that mPEG-*b*-PBE_36_ micelles not only promote tumor cell suppression but also significantly enhance the induction of apoptosis in melanoma cells compared to the untreated and BNCT-only treated groups. The marked efficacy of mPEG-*b*-PBE_36_ micelles in activating key biomarkers of tumor suppression and apoptosis underscores their potential as superior boron delivery agents for BNCT.

## Conclusions

In conclusion, we have successfully synthesized a well-defined mPEG-*b*-PBE_36_ block copolymer with a high boron content via the ATRP technique. The self-assembly of these block copolymers resulted in the formation of micelles with an average diameter of 43 ± 10 nm, which is within the ideal nanotherapeutic size range (30–200 nm) for drug delivery applications. Our investigations highlighted the critical role of initial polymer concentration in influencing the size, morphology, stability, and yield of the polymer micelles. To address the challenges of aggregation, swelling, and even hydrolysis that polymer micelles encounter during storage in an aqueous environment, this study also delved into various reconstitution techniques following the lyophilization of the polymer micelles. Probe sonication for a duration of 30 s emerged as the most effective technique, yielding uniformly sized and well-dispersed spherical micelles. In vitro assay utilizing melanoma cells revealed exceptional biocompatibility of mPEG-*b*-PBE_36_ micelles, maintaining cell viability above 92% at concentrations up to 1000 μg/mL over 72-h period. The cellular uptake efficiency of these micelles, as accessed through ICP-MS analysis and confocal imaging, demonstrated a remarkable 38-fold enhancement in boron accumulation compared to BPA after 6 h of incubation. Subsequently neutron irradiation of melanoma cells treated with mPEG-*b*-PBE_36_ micelles resulted in over 30% cell apoptosis, markedly higher than approximately 10% observed in BPA-treated cells after 48 h of irradiation. In vivo biodistribution study conducted on a B16F10 melanoma mouse model revealed 2-fold enhancement in tumor accumulation of mPEG-*b*-PBE_36_ micelles compared to the BPA-treated group. Notably, mice treated with the mPEG-*b*-PBE_36_ micelles and subjected to neutron irradiation exhibited significant tumor growth inhibition with a TGD of 5.01 days, surpassing the TGD observed in the BPA group (TGD = 3.38 days). Immunohistochemistry staining further validated the enhanced antitumor efficacy of the mPEG-*b*-PBE_36_ micelles in comparison to BPA after BNCT treatment, affirming their potential as effective boron carriers for BNCT. In summary, this work presents the first in vivo proof-of-concept demonstration of boron-rich, size-tunable, nontoxic, mPEG-*b*-PBE_36_ polymer micelles as promising candidates for boron delivery in BNCT, marking a significant advancement in field of cancer therapy.

## Supporting Information Available

*M*_n_ of the block copolymers with varying degrees of polymerization, hydrodynamic size distribution of mPEG-*b*-PBE_36_ micelles, micelle stability in PBS and in DMEM, and the parameter and tumor growth delay (PDF)
