# Supplementary material for: Boron Neutron Capture Therapy Enhanced by Boronate Ester Polymer Micelles: Synthesis, Stability, and Tumor Inhibition Studies
Source: Biomacromolecules. 2024 Jun 7;25(7):4215–32. doi: 10.1021/acs.biomac.4c00298 (PMC11238341; doi:10.1021/acs.biomac.4c00298)
Supplement: Supplementary file 1 — bm4c00298_si_001.pdf [file bm4c00298_si_001.pdf]

Boron Neutron Capture Therapy Enhanced by Boronate Ester Polymer Micelles: Synthesis, Stability, and Tumor Inhibition Studies.

*Wan Yun Fu, Yi-Lin Chiu, Shi-Chih Huang, Wei-Yuan Huang, Fang-Tzu Hsu, Han Yu Lee, Tzu-Wei Wang, Pei Yuin Keng**

Correspondence: keng.py@mx.nthu.edu.tw

Department of Material Science and Engineering, National Tsing Hua University, Hsinchu City 300, Taiwan.

KEYWORDS. Polymer micelles, boron neutron capture therapy, drug delivery, cancer treatment, lypophilization,

Supporting information

Table S1. The DP and M_n of the mPEG-b-PBE amphiphilic block copolymers obtained from end-group analysis via ^1H NMR. The different DP and M_n were achieved by varying the [M]/[I] ratio using CuBr as catalyst and PMDETA as ligand.

DP	Molar ratio of Mbpin and mPEG-Br	Reaction time	$M_{n,NMR}$ (g mol^{-1})
27	1:50	12 hr	11,371
36	1:50	24 hr	13,441
78	1:100	12 hr	23,101
103	1:100	24 hr	28,851

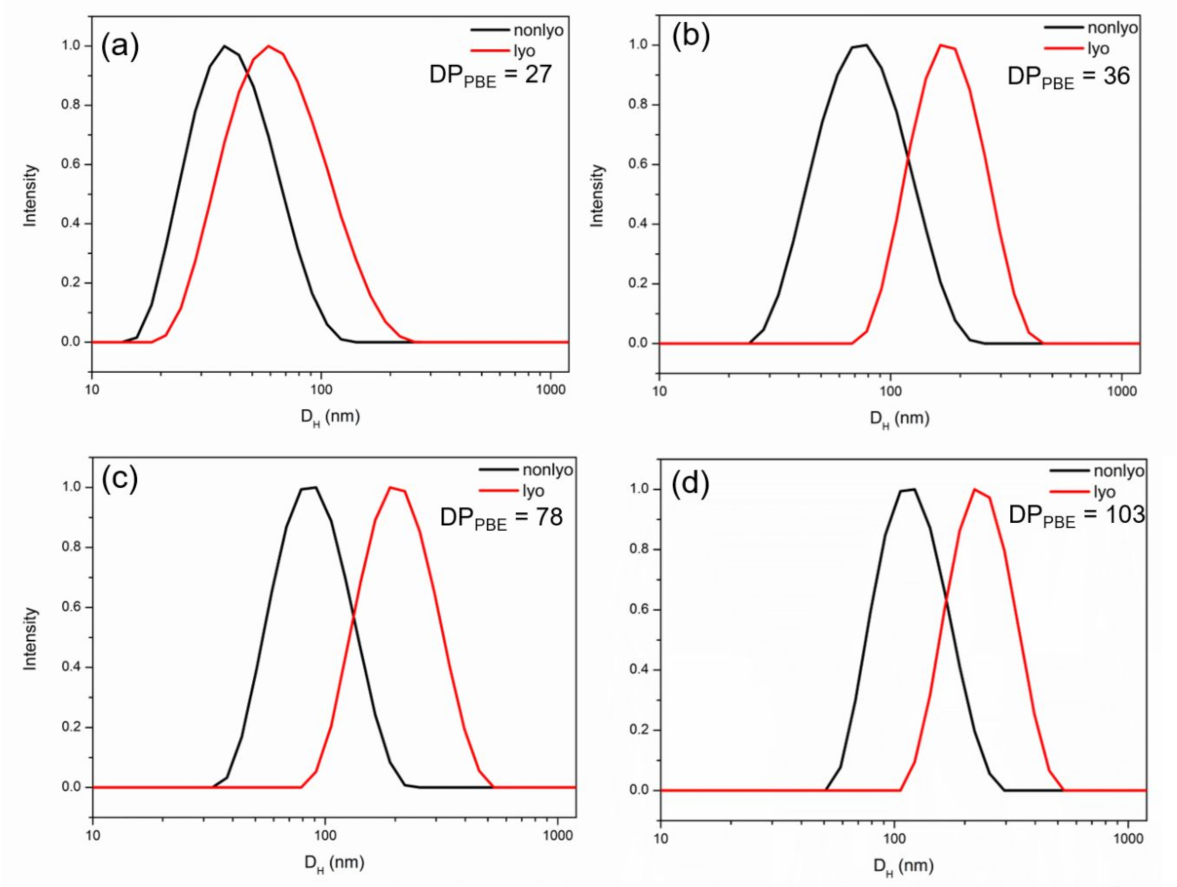

Figure S1. Hydrodynamic size distribution of mPEG-b-PBE$_{36}$ micelles prepared using Method 1 with varying degree of polymerization (DP) of PBE. DLS traces are represented by number distribution: nonlyophilized micelles are depicted in black traces, and lyophilized micelles in red traces. Panel (a) shows micelles with DP$_{PBE}$ = 26, (b) DP$_{PBE}$ = 36, (c) DP$_{PBE}$ = 78 and (d) DP$_{PBE}$ = 103.

Table S2. The stability of mPEG-b-PBE$_{36}$ micelles incubated in PBS and DMEM over 7 days. The hydrodynamic size of mPEG-b-PBE$_{36}$ were determined by DLS.

Block copolymer	day	d[a] (in PBS) (nm)	PDI[b] (in PBS)	d[c] (in DMEM) (nm)	PDI[d] (in DMEM)
mPEG-b-PBE_{36}	1	66	0.24	51.4	0.34
	3	69	0.23	76.7	0.20
	7	67.5	0.14	99.9	0.17

[a] Average dimension of the micelles incubated in PBS measured by DLS.

[b] Polydispersity of micelles incubated in PBS measured by DLS.

[c] Average dimension of the micelles incubated in DMEM measured by DLS.

[d] Polydispersity of micelles incubated in DMEM measured by DLS.

Table S3. The parameters and tumor growth delay (TGD) of the B16F10 melanoma mice under different treatment.

**Treated drug**	α	β	γ	**DT**	**TGD**
**Control**	5.32	0.409	-	1.69	-
**Control (BNCT)**	5.31	0.32	5.5	2.19	0.49
**BPA (BNCT)**	5.55	0.146	5.43	5.57	3.38

**mPEG-b-PBE micelles (BNCT)**	5.46	0.106	5.43	6.71	5.01
